# Caregiver perceptions of nutrition interventions in infants and children under 24 months of age: a systematic review

**DOI:** 10.1017/S1368980023001246

**Published:** 2023-09

**Authors:** Isabella Stelle, Mai-Lei Woo Kinshella, Sophie E Moore

**Affiliations:** 1 Department of Women and Children’s Health, King’s College London, St Thomas’ Hospital, Westminster Bridge Rd, London SE1 7EH, UK; 2 Department of Obstetrics and Gynaecology and BC Children’s Hospital Research Institute, University of British Columbia, Vancouver BC, Canada; 3 Medical Research Council Unit The Gambia at The London School of Hygiene and Tropical Medicine, Fajara, The Gambia

**Keywords:** Nutrition interventions, Infants, Caregivers, Acceptability, Qualitative

## Abstract

**Objective:**

Efficacy studies show early nutrition interventions improving infant nutrition status, but understanding caregiver acceptability is required for implementation of such interventions. This systematic review examines caregivers’ perceptions of nutrition interventions in young children.

**Design:**

We searched the Cochrane Central Register of Controlled Trials, MEDLINE, Embase, CINAHL and PsychINFO from date of online journal inception through December 2020. Interventions included oral (powder/liquid/tablet) and/or intravenous supplementation, food fortification and nutrition counselling. Inclusion criteria included primary research, data presented on caregiver perception and studies published in English. Quality assessment was performed using the Critical Appraisal Skills Programme tool. Studies underwent narrative synthesis using inductive thematic analysis.

**Setting:**

No restriction.

**Participants:**

Caregivers of children under 24 months of age.

**Results:**

Of 11 798 records identified, thirty-seven publications were included. Interventions included oral supplementation, food fortification and nutrition counselling. Caregivers included mothers (83 %), fathers, grandparents and aunts. Perceptions were gathered through individual interviews, focus group discussions, questionnaires, surveys and ratings. Totally, 89 % of studies noted high acceptability (*n* 33 most notably increased appetite (*n* 17). In total, 57 % of studies (*n* 21) cited low acceptability, commonly from side effects (*n* 13) such as gastrointestinal issues, appetite loss and stained teeth.

**Conclusions:**

Positive perceptions and enthusiasm for interventions were frequently reported. Key to implementation was the increased appetite noted by caregivers. A substantial proportion of studies reported negative perceptions, mainly due to side effects. In future interventions, mitigation and education around common side effects are crucial for acceptability. Understanding both positive and negative caregiver perceptions is important for informing future nutrition interventions and strengthening sustainability and implementation.

In 2019, an estimated 5·2 million children under 5 years of age died mostly from preventable and treatable causes^(1)^. Nutrition-related factors contributed to approximately 45 % of these deaths^(1)^. Further, undernutrition during the first 1000 d of life (from conception until 2 years of age) can have lifelong consequences for growth and cognitive development^(2)^.

The first 1000 d of life are an especially sensitive period due to rapid growth and development, increased nutritional needs, greater vulnerability to infection and full dependency for care^(2)^. Evidence has shown that early years are critical for cognitive, language and social-emotional development with risks for poor development being linked to inadequate quality of caregiver to child interaction^(3)^. Caregiver dependency at this young age is therefore vital in determining nutritional adequacy. For example, between the age of 6–23 months, in areas with an Fe deficiency anaemia prevalence greater than 40 %, the WHO recommends daily Fe supplementation (10–12·5 mg elemental Fe) for three consecutive months per year^(4)^. However, Fe supplementation is commonly noted to cause adverse gastrointestinal side effects in children such as diarrhoea, nausea, constipation and vomiting, therefore limiting adherence in supplementation trials (170). More recent studies have shown Fe-containing micronutrient powders to cause increased risk of diarrhoea in young children (162, 167, 172, 173). Therefore, a proposed intervention must be perceived as acceptable through the caregiver’s perspective for effective implementation, given care providers are the ones administering interventions to their infants and young children. Efficacy studies in infants have shown that early nutrition interventions improve infant nutrition status, but a better understanding of caregiver acceptability is required to examine whether such interventions can be effectively taken to scale^(5–7)^.

This review explores caregivers’ perceptions of various nutrition interventions within a global context for infants and young children under 24 months of age as well as reported side effects and impacts on infant feeding practices.

## Methods

The primary objective of this review is to examine caregiver perception of nutrition interventions (i.e. oral (powder, liquid, tablet) and/or intravenous supplementation, food fortification and nutrition counseling) in their infants under 24 months of age. Further, secondary objectives include a consideration of the effects of nutrition interventions in early infancy (the first 24 months of life) on choices of infant feeding practices and any adverse effects of nutrition interventions in early infancy (the first 24 months of life).

We searched the Cochrane Central Register of Controlled Trials (CENTRAL), MEDLINE (Ovid), Embase (Ovid), CINAHL and PsychINFO from date of online journal inception through December 2020. Searches were supplemented by scanning reference lists of papers included for review. Based on the PICOS research framework (Table 1), search terms were developed (Table 2). A review protocol detailing the research question, search strategy, inclusion and exclusion criteria, quality assessment and strategy for data synthesis was developed to refine the scope of the review. The protocol was registered to Prospero (CRD42021238050). Study selection was managed using Covidence^(8)^. Titles and abstracts were manually screened according to the eligibility criteria (Table 3) by two independent reviewers (IS and MWK). Discrepancies were resolved by discussion and a third reviewer (sem) adjudicated in the absence of consensus. Full texts were then reviewed by the two independent reviewers (IS, MWK). The third reviewer (sem) provided an independent assessment in any disputes regarding eligibility.

**Table 1:** PICOS research framework

Population	Caregivers of infants under 24 months of age (i.e. mothers, fathers, mothers-in-law, grandmothers and family members)
Intervention	Nutrition interventions (oral (powder, liquid, tablet) and/or intravenous supplementation, food fortification and nutrition counselling)
Comparison	NA, placebo, no intervention/therapy and additional nutrition interventions
Outcome	Perception of intervention (secondary: impact on exclusive breast feeding, feeding practices and adverse events)
Study design	Experimental studies (controlled trials) and observational studies (cohort, case controlled, cross-sectional and qualitative)

**Table 2:** Search terms

Searches	Search term
Population	Caregiver/ Mothers/ (mother* or maternal*).ab. Fathers/ (father* or paternal*).ab. Parents/ Family/ (grand?parent* or grand?mother* or grand?father*).mp.
Intervention	Infant/ or Infants/ or Infancy/ exp Infant, Newborn/ child/ (infant* or newborn* or new born* or neonat* or baby or babies or child* or young children).ti,ab. Micronutrients/ or Trace Elements/ Dietary Supplements/ (micronutrient* or multinutrient* or multimicronutrient* or multivitamin* or multimineral* or MMN* or MMS* or MNP* or sprinkles or LNS*).tw. (trace adj (element* or mineral* or nutrient*)).tw. (diet* adj3 supplement*).tw. Nutrition.mp. (diet* counselling or nutrit* counselling or lactation counselling or breast?feeding counselling).mp. (fortification or fortified foods).mp.
Outcome	Qualitative Research/ or Interviews as Topic/ (qualitative or group discussion? or focus group? or themes).ti,ab. Perception/ Implementation/ (acceptab* or feasib* or program evaluation).tw. (barrier* or facilitator* or like* or dislike*).mp.

**Table 3:** Eligibility criteria

Selection criteria	Inclusion	Exclusion
Population	Caregivers; average age of infants < 24 months of age	Average age of infants > 24 months of age
Intervention	Oral (powder, liquid and tablet) and/or intravenous supplementation, food fortification and nutrition counselling	No nutrition intervention
Comparison	Placebo, no intervention/therapy, oral (powder, liquid and tablet) and/or intravenous supplementation, food fortification, nutrition counselling	
Outcome	Caregiver perception of intervention	No caregiver perception of intervention
Study type	Published experimental and observational studies in English including randomised or non-randomised controlled trials, cohort, case controlled, cross-sectional survey, facility evaluations and qualitative studies	Studies without primary data collection, such as reviews and study protocols, studies not in English and those not demonstrating a clear research methodology (i.e. abstracts, conference proceedings, commentaries, letters and editorials)
Language	Studies published in the English language	Not in English
Other	From conception of the journal through 2020	Published after 2020

Inclusion and exclusion criteria can be found in Table 3. The primary outcome was the caregivers’ perceptions of the intervention, and secondary outcomes included the impact on exclusive breast feeding, feeding practices and/or adverse events. All caregiver perceptions were considered and considered of equal importance. Secondary outcomes were summarised in the narrative synthesis.

Quality assessment was performed for all included studies using the Critical Appraisal Skills Programme tool^(9)^. Critical Appraisal Skills Programme is the most used tool for quality appraisal in health-related qualitative evidence syntheses, with endorsement from the Cochrane Qualitative and Implementation Methods Group^(10,11)^. Quality assessment was reported for all identified studies to inform interpretation. Due to the exploratory nature of qualitative studies, no studies were excluded based on the quality assessment.

A data extraction sheet was developed and piloted by the research team. Details about the nutrition intervention, study methodology, sample size, study design and outcomes (perceptions) were extracted into Excel^(12)^. Two reviewers (IS, MWK) independently extracted data and conducted the quality assessment from a sample of eligible studies (10 %) until agreement was achieved, with the remainder extracted by one reviewer (IS). The data extraction sheet was imported into NVivo where inductive thematic analysis of caregiver perception was conducted^(13,14)^. Excerpts from included studies were extracted to generate key themes^(14)^. Themes are repeated patterned responses within data sets that are then separated into sub-themes^(14)^. An inductive approach was used in which the data analysis is data driven so that the participant’s views take precedence^(14)^.

The Preferred Reporting Items for Systematic Review and Meta-analysis (PRISMA) guidelines are used^(15)^. The PRISMA Checklist is presented in online supplementary material, Supplemental Extended Datafile 1.

## Results

### Characteristics of included studies

**Fig. 1:**
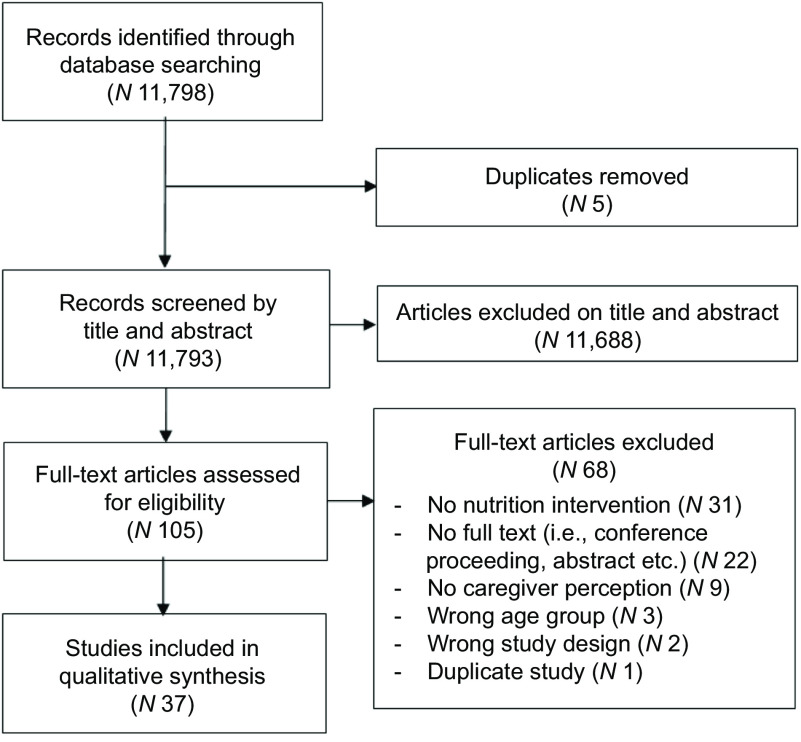
PRISMA flowchart

Of 11 798 records identified, thirty-seven publications were included (Fig. 1). One hundred and five full-text articles were reviewed for eligibility. Of these, thirty-one were excluded because they used no nutrition intervention, twenty-two were not full texts (i.e. conference proceeding, abstract, etc.), nine had no caregiver perceptions reported, three were in the wrong age group, two were the wrong study design (i.e. unapplicable intervention) and one was a duplicate (see online supplementary material, Supplemental Extended Datafile 2).

**Fig. 2:**
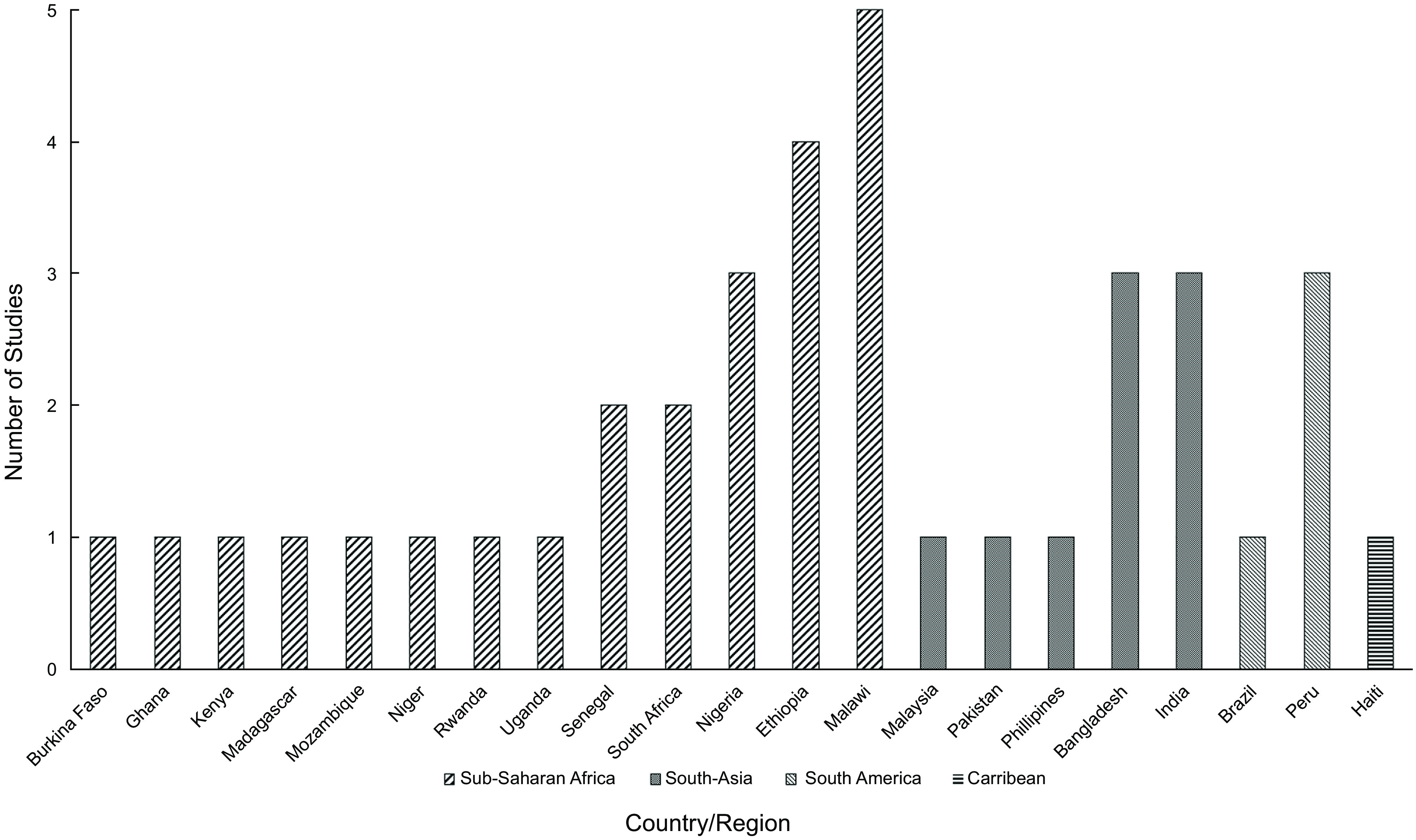
Spread of countries included in this review

Despite no restriction on publication start date, all thirty-seven studies were published between 2009 and 2020 (see online supplementary material, Supplemental Extended Datafile 3a for characteristics of included studies). Sample sizes varied widely from eleven up to 1916 caregivers with data from 13 765 caregivers in total across the included studies. The data collection methodology gathered perceptions through interviews, focus group discussions, questionnaires, surveys and ratings. The scope of caregivers included mothers (*n* 11 466), fathers (*n* 370), maternal and paternal grandparents (*n* 20), aunts (*n* 4) and unspecified (*n* 1905). The nutrition interventions included small quantity lipid-based nutrient supplements, ready to use supplementary foods, micronutrient interventions in different formulations (Fe folic acid syrup, multiple micronutrient powders and micronutrient powders), lipid-based nutrient supplements, ready to use therapeutic foods, oral rehydration solutions, infant and young child feeding practices, social and behavioural change communications, nutrition counselling, complementary foods, fortified porridges and crops and food grinders. Of the geographical regions represented, twenty-four of the studies were in sub-Saharan Africa (one was a dual country study, both in sub-Saharan Africa), nine in South-Asia, four in South America and one in the Caribbean (Fig. 2). Despite no limitations on setting, all studies took place in low-resource settings.

### Quality assessment

For quality appraisal, of the thirty-seven publications, two-four ranked ‘good’, twelve ranked ‘fair’ and one ranked ‘poor’. The one study that ranked as ‘poor’ did not specify its recruitment strategy, the relationships between the researcher and the participants or how the data was analysed^(16)^. The full results from the Critical Appraisal Skills Programme tool can be found in online supplementary material, Supplemental Extended Datafile 4.

### Themes and sub-themes

**Fig. 3:**
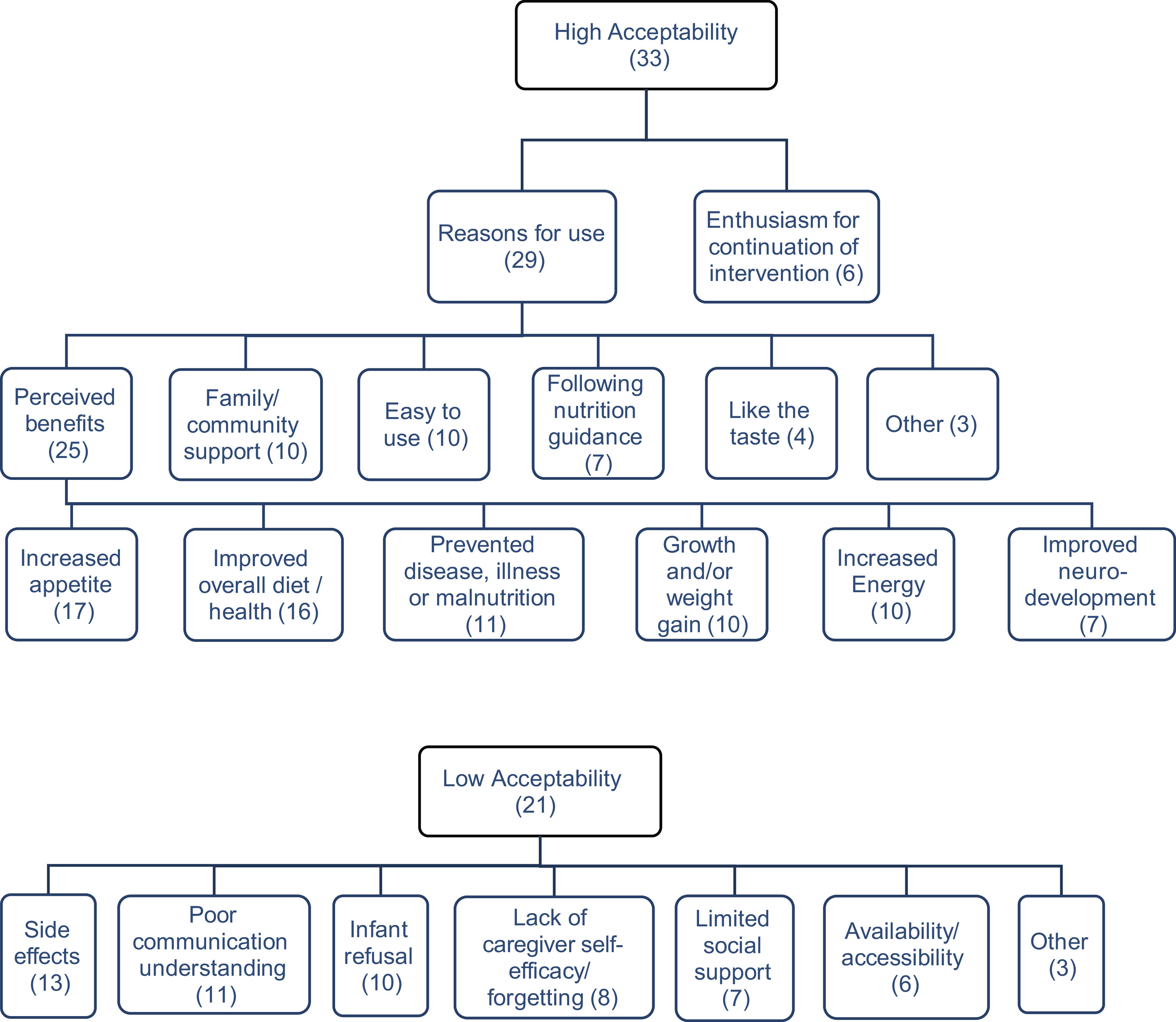
Acceptability of interventions

From the inductive thematic analysis of caregiver perception, two main themes emerged: high and low acceptability (Fig. 3). For high acceptability the following sub-themes emerged: reasons for use (*n* 29) and enthusiasm for continuation of the intervention (*n* 6). ‘Reasons for use’ of the intervention was further broken down, specifically into sub-themes around its use for ‘perceived benefits’ to the infant. For low acceptability, seven sub-themes of reasons for low acceptability emerged. These sub-themes included side effects (*n* 13), poor communication/ understanding (*n* 11), infant refusal (*n* 10), lack of caregiver self-efficacy/forgetting (*n* 8), limited social support (*n* 7), availability/accessibility (*n* 6) and other (*n* 3). Nineteen of the studies cited both low and high acceptability.

### High acceptability

Eighty nine percent of studies noted high acceptability (*n* 33; detailed in online supplementary material, Supplemental Extended Datafile 3b), with six mentioning enthusiasm for continuation of the intervention.

#### Perceived benefits

The most noted perceived infant benefits (*n* 25) were increased appetite (*n* 17), improved overall health and diet (*n* 16) and prevention of disease, illness or malnutrition (*n* 11). When noting improved overall diet and health, specifics of perceived increased blood health and digestion were highlighted^(17–21)^. In the context of prevention of disease, illness or malnutrition, the interventions were compared with a medicine in a beneficial sense^(22–25)^. Additionally, caregivers noted perceptions of improved growth (height and weight) and improved strength (*n* 10), as well as improved energy (*n* 10) and neurodevelopmental improvements (*n* 7)^(17–21,24–32)^.

#### Other facilitators of high acceptability

Other facilitators associated with high acceptability: family and community support, ease of use, following nutrition guidance, palatability and others. A significant and important facilitator for high acceptability was family and community support (*n* 10)^(18,21,25,29,31,33–37)^. Within the context of patriarchal households, when mothers felt supported by their husbands, they were more inclined to adhere to an intervention^(21,31,33,35–37)^. Ease in packing and storage, accessibility and affordability were keys factors in caregiver use of intervention (*n* 10)^(20,24–26,29,31,35,38–40)^. Education around purpose and administration of the intervention were key to ensuring acceptability and continuation of use as caregivers wanted to follow nutrition guidance when it was given (*n* 7)^(18,19,22,25,28,33,41)^. It was noted that giving the intervention to their infant gave caregivers a feeling of empowerment^(33)^. Having an infant like the taste also increased the chances a caregiver continued the interventions use (*n* 4)^(24,26,31,42)^.

One study found that mothers preferred clinic administration of the supplements as it was perceived as ‘more hygienic’ and ‘involving professional health workers’^(43)^. However, other mothers, especially those living further from the clinics, preferred house-to-house delivery of the supplements, which is the traditional method for a nutrition campaign in their region^(43)^.

### Side effects

Side effects that were noted, but not in the context of low acceptability were diarrhoea, vomiting, and constipation, nausea, lack of appetite and abdominal discomfort, respectively, from most to least cited^(21,23,27,32,36,37,44)^.

### Low acceptability

Just over half of the studies (*n* 21) cited low acceptability (detailed in online supplementary material, Supplemental Extended Datafile 3b). This was most commonly due to side effects (*n* 13), poor communication and/or understanding (*n* 11) and infant refusal (*n* 10).

#### Side effects

Side effects were noted in thirteen studies^(17,18,20,21,23,25,27,29,34,37,44–46)^. All thirteen studies had caregivers reporting these side effects as negative; however, they were also noted as, when mild, a sign of the intervention being effective. For example, one mother noted her infants’ change in stools as an indication that the intervention was working^(34)^. Side effects that led to low acceptance were noted as the infant being hyperactive, diarrhoea, vomiting, constipation, abdominal discomfort, loose or black stools, infant rejection, appetite loss, stained teeth, the infant getting sick, a decrease in overall health, general negative effect and in one Peruvian peri-urban community, a mother citied impaired mental development as a concern^(21)^.

#### Other barriers to intervention acceptability

When there was lack of caregiver self-efficacy and forgetfulness (*n* 8), limited social support (*n* 7) and/or availability/accessibility to the interventions (*n* 6), these were significant barriers to caregiver lack of use. Other reasons for non-use (*n* 3) were simply that caregivers had no reason as to why they did not want to use it and because they were using another medication at the time instead so stopped the intervention^(17,26)^. One study noted a high prevalence of no impact from the intervention (20 %)^(20)^.

### Nutritional Intervention preferences

Among studies that compared acceptability of various supplements, the following preferences were found. Mothers noted that micronutrient powders *v* Fe syrups were better suited for older children who had ‘*become smart*’ and were able to eat on their own^(32)^. However, they felt that the syrups were better in ensuring the infant received the full dose of the supplement because with powders the infant did not always finish their food^(32)^. Overall, the enthusiasm for continued use of syrups *v*. powder was equal (38 % each) and 24 % of mothers said they would like to use both^(32)^. Additionally, caregivers found it easier to give micronutrient powders in semi-solid foods such as purees and porridges rather than liquid preparations such as soups^(21)^. When looking at complementary foods fortified with or without Zn, those without Zn scored higher on a degree of liking scale^(47)^. Novel rice-lentil- and chickpea-based ready-to-use supplementary foods rating significantly better for ‘overall liking’ when compared with traditional Puschti packet^(16)^. In terms of frequency of supplementation, different studies found varying preferences for flexible verses daily verses every other day administration. For mothers giving flexible or daily administration, almost all mothers preferred flexible due to perceived benefits such as more time to give the Sprinkles, more autonomy and less anxiety around missing a dose^(48)^. Alternatively, two studies found that caregivers preferred daily administration of micronutrient powders verses alternate days, so they were less likely to forget^(20,29)^. While not in a negative context, mothers also reported, organoleptic changes in food, such as colour, taste, smell and texture, when certain interventions, such as powders, were added to home foods^(29,37)^.

### Misuse of supplements

Misuse of supplements was seen in seven studies through sharing with other infants or giving more to the infant when it was demanded^(18,23,24,26,29,30,49)^. Likewise, jealousy among other households or household members, as well as theft of supplements, posed an issue^(22,23,49)^.

### Impact on feeding practices

Secondary objectives included a consideration of the effects of nutrition interventions in early childhood (the first 24 months of life) on choices of infant feeding practices; however, this theme was not seen in any of the included studies.

## Discussion

The purpose of this review was to understand caregiver perceptions around nutrition interventions for use in infants and young children. This is especially relevant in low-resource settings where caregiver acceptability of nutrition interventions, alongside appropriate infant feeding practices, may be critical for both short- and long-term health.

Interestingly, all the studies identified took place in low-resource settings with the majority being in sub-Saharan Africa. Previous literature has found that low- and middle-income countries are unrepresented in clinical research^(50)^, specifically with the number of clinical trials in sub-Saharan Africa being significantly less in proportion to the disability adjusted life years present^(51)^. Understanding caregiver perceptions of nutrition interventions is of global relevance, especially in low-resource settings.

A significant emergent theme was the importance of family and community support^(18,21,25,29,31,33–37)^. Concurrent with the literature, mothers were by far the most cited caregiver^(52)^, so in the context of patriarchal households, when the mothers felt supported by their husbands, they were more inclined to adhere to an intervention^(21,31,33,35–37)^. It has been previously found that to ensure acceptance, education played a significant role in caregivers’ continuation of use^(53,54)^. Caregivers wanted to follow nutrition guidance when it was given but understanding of why the intervention was needed was pertinent. It was noted that giving the intervention to their infant gave caregivers a feeling of empowerment as they knew they were doing something good for their infants^(33)^. One of the most noted reasons for low acceptability was poor communication and/or understanding such as lack of caregiver self-efficacy and forgetfulness, limited social support and/or availability/accessibility to the intervention. Appropriate training of intervention use is essential; in one study, a participant reported stopping the intervention when the infant needed medication, counter to the intention of the study^(27)^. One caregiver simply gave no reason, they just did not want to use the intervention, and another cited seeing no impact from the intervention^(17,20,26)^. Education around interventions not always having an immediate impact, but rather long-term benefits may have solved this issue. Lastly, ease of use of packaging and storing, as well accessibility and affordability, was indicated as crucial^(20,24–26,29,31,35,38–40)^.

Almost all the studies noted high acceptability. Instances of misuse were common due to the infants demanding more of the supplement^(18,23,24,26,29,30,49)^ or jealously from other family or community members which lead to sharing^(22,23,49)^. In these communities, feeding is often done communally so sharing can be difficult to avoid, as has been seen in previous studies^(55,56)^. The most frequently cited reason for this high acceptability was an increase in the infant’s appetite. While improved overall diet and health were almost equally as noted, interestingly a few caregivers specifically noted an increase in the infant’s ‘*blood health*’. Also highly cited was the interventions’ ability to prevent disease, illness and malnutrition, with the intervention even being compared with medication^(22–25)^, as similarly reported in previous studies^(55,57)^. Interestingly, when noting improved growth, such as weight, height and strength, some caregivers also noted neurodevelopmental improvements^(17–21,24–32)^. Caregivers did report organoleptic changes in food (colour, taste, smell and texture) when micronutrient powders were added^(29,37)^. Having the infant like the taste was important in continuation of the intervention^(24,26,31,42)^. One of the most common reasons for low acceptability was when the infant refused the intervention.

Low acceptability was most commonly due to side effects, such as such as reports of infants’ morbidity, including diarrhoea. However, side effects were not always noted in the context of low acceptability, but rather a result of the intervention and even sometimes as a sign that the intervention was working, in the case of darkening of stool^(34)^. As previously noted in the literature, side effects are common with nutrition interventions at this young age, especially in those containing Fe^(58)^. This difference in reporting side effects highlights the issue of heterogeneity in studies such as these. Given caregivers perceptions are being collated, this could simply reflect a difference in caregiver attention and sensitivity to their child’s reaction.

It was evident that personal preferences varied, with nineteen of the studies citing both low and high acceptability. This is important to take into consideration with interventions, but heterogeneity in the studies makes it difficult to form conclusions for general recommendations. For example, some mothers preferred clinic administration because it was seen as more hygienic, whereas some mothers preferred home administration because it was more convenient^(43)^. Additionally, age may impact what type of intervention should be given to children, with caregivers noting that syrups were better for infants to ensure the whole dose was received but that powders were better for older children who were able to eat on their own^(32)^. Additionally, it was found that micronutrient powders were better suited for semi-solid foods such as purees and porridges^(21)^. Likewise, and as debated in previous literature, when it comes to frequency of the interventions, preferences were divided across daily or flexible administration^(59)^. Some caregivers preferred daily as it became a routine and they were less likely to forget^(20,29)^, whereas other caregivers preferred flexible administration thanks to more autonomy and less worry about missing a dose^(48)^. Additionally, misuse of supplements, such as using supplements for other family members, could be associated with variations in positive verses negatives responses, which highlights the importance of appropriate counselling of family members around supplements and their use.

While negative perceptions were reported, overall positive perceptions and enthusiasm for continuation of the interventions were more frequent. Finally, though there were preferences expressed for type of supplementation, route, dosing, administration and packaging, these preferences were not frequent enough to draw conclusions. Caregiver perception is crucial for future acceptability, adherence to and effectiveness of interventions. As evident from the results, future interventions can best support this through education as well as family and caregiver support.

The main strength of this systematic review is that is it the first comprehensive review of caregiver perception of nutrition interventions in infants and young children. In addition, trials were not excluded based on supplementation regimen allowing the authors to understand a wide scope of the literature in the field. All studies, but one, were assessed to be good or fair quality, which supports the credibility of review findings.

We also acknowledge several limitations. While heterogeneity in the data was a strength in terms of assessing the full scope of the literature, it makes comparison of trials via a quantitative meta-analysis difficult. Most studies also cited both high and low acceptability, so it was difficult to categorise an intervention as acceptable or not. Likewise, conclusions for specific interventions were hard to draw due to the limited number of studies. While there was no start date limitation (journal induction), the first study included in this review was from 2009. Due to implementation research being a newer science, the research in this field is still limited. Some of the studies used focus group discussions and interviews, which gave useful insights into caregiver perceptions, but many studies simply using ratings, surveys and questionnaires. While still useful, these studies lacked as much depth as those using more personal research practices. Additionally, some studies did not specify who the caregivers were.

### Conclusion

Positive perceptions and enthusiasm for continuation of interventions were frequently reported by caregivers. Key facilitators to implementation were increased appetite and overall general health of infants and children as noted by caregivers. A substantial proportion of studies reported negative perceptions, mainly due to side effects. In future interventions, mitigation and education around common side effects are crucial for acceptability. Caregivers wanted to follow nutrition guidance when it was given, but understanding of why the intervention was needed was pertinent as one of the most noted reasons for low acceptability was poor communication and/or understanding around the intervention. Likewise, cultural circumstances must be taken into consideration, for example, feeding is often done communally in low-resource communities so sharing can be difficult to avoid. Additionally, having the supplement be palatable is important as one of the most common reasons for low acceptability was when the infant refused the intervention. Continued research is needed in this area, especially in low-resource settings where nutrition deficiencies are common in infants and can have lifelong impacts. An understanding of the barriers and facilitators to implementing such interventions, especially through the perception of caregivers, is crucial for the success of future interventions.

## Supplementary material

For supplementary material accompanying this paper visit https://doi.org/10.1017/S1368980023001246. click here to view supplementary material
