# Supplementary material for: Caregiver perceptions of nutrition interventions in infants and children under 24 months of age: a systematic review
Source: Public Health Nutr. 2023 Jun 23;26(9):1907–16. doi: 10.1017/S1368980023001246 (PMC10478058; doi:10.1017/S1368980023001246)
Supplement: Supplementary file 1 [file S1368980023001246sup.zip › S1368980023001246sup001.docx]

**Extended Datafile 3a.** Characteristics of studies included in this review

| **Reference** | **Country** | **Data Collection Methodology** | **Caregiver** | **Sample size** | **Nutrition intervention** |
| --- | --- | --- | --- | --- | --- |
| Aaron et al, 2011 | Senegal | Ratings | Caregiver | 21 | Complementary foods (CF) fortified with iron or the same level of iron + zinc from 12 months (mo) of age |
| Adams et al, 2018 | Ghana | Surveys | Mothers and fathers | 1,273 | Small quantity lipid-based supplements (SQ-LNS) from six mo of age |
| Ahmed et al, 2014 | Bangladesh | Questionnaires and ratings | Mothers/ caregivers | 90 | Three ready-to-use supplementary food (RUSFs): rice-lentil based, chickpea based and standard RUSF (Pushti packet cereal-based) from six mo of age |
| Andersen et al, 2009 | South Africa | Focus group discussions (FGDs) | Mothers | 28 | Nutrition Supplementation Programme: rehabilitation program for undernourished children under five years of age |
| Angdembe et al, 2015 | Bangladesh | Questionnaires | Mothers | 78 | Multiple micronutrient powders (MMNPs) from six months of age |
| Ashorn et al, 2015 | Malawi | Knowledge, attitudes, and practices (KAP) interviews and in-depth interviews (IDIs) | Mothers | 1,098 | Lipid-based nutrient supplements (LNS) from six months of age |
| Athavale et al, 2020 | India | IDIs | Mothers and paternal grandmothers | 33 | Infant and young child feeding (IYCF) counselling from six mo of age |
| Bashir et al, 2016 | Pakistan | Questionnaires | Mothers | 60 | Ready to use therapeutic food (RUTF) for malnourished children from three mo of age |
| Brewer et al, 2020 | Peru | IDIs and FGDs | Mothers, fathers, grandparents, and aunts | 129 | Micronutrient powders (MNPs) from six mo of age |
| Campos et al, 2015 | Brazil | Ratings | Mothers | 50 | Homemade chicken liver baby food versus ground beef baby food (control) from six mo of age |
| Cohuet et al, 2012 | Niger | IDIs, FGDs, and questionnaires | Caregivers | 1,842 | RUSF from six mo of age for infants with moderate acute malnutrition (MAM) or at risk of malnutrition |
| Creed-Kanashiro et al, 2016 | Peru | IDIs | Caregivers | 64 | MNPs for anaemic children from six mo of age |
| Creed-Kanashiro et al, 2018 | Peru | IDIs | Mothers | 108 | Commercial infant food grinders where there is delayed/low consumption of thick CFs, including animal-source foods from six mo of age |
| Goyena et al, 2019 | Philippines | IDIs | Mothers | 141 | MNPs from six mo of age |
| Gunaratna et al, 2015 | Ethiopia | Questionnaires and ratings | Mothers | 61 | A biofortified crop, quality protein maize (QPM), as a CF versus conventional maize from six mo of age |
| Hess et al, 2011 | Burkina Faso | Ratings | Mothers | 36 | LNS with or without zinc from nine mo of age (breastfed) |
| Horton et al, 2018 | Senegal | FGDs | Mothers and grandmothers | ~64-96 | Government vitamin A distribution from six mo of age |
| Ip et al, 2009 | Bangladesh | FGDs | Mothers | 54 | Sprinkles from six mo of age |
| Jefferds et al, 2010 | Kenya | FGDs | Mothers and grandmothers | ~30-54 | Sprinkles from six mo of age |
| Kajjura et al, 2020 | Uganda | FGDs and IDIs | Mothers | 156 | Supplementary food (SF) in children with MAM using either a malted sorghum-based porridge or fortified corn soy blend as a porridge from six mo of age |
| Kodish et al, 2017 | Mozambique | IDIs | Male and female caregivers | 31 | SQ-LNS from six mo of age |
| Korenromp et al, 2015 | Nigeria | Surveys and questionnaires | Caregivers (Mothers) | 272 | Health service MNPs from six mo of age |
| Kung’u et al, 2015 | Nigeria | Semi-structured interviews | Caregivers | 1,195 | Nutrition training of oral rehydration solution (ORS) for use in diarrhoea followed by a take home diarrhoea management kit (zinc + ORS) for children under five years of age |
| Kwon et al, 2014 | India | Semi-structured interviews and FGDs | Mothers and other caregivers | 38 | Nutrition education on the use of daily oral ferrous sulphate for children under five years of age |
| Locks et al, 2017 | Madagascar | Surveys | Mothers | 1,204 | Integrated IYCF-MNP program aimed to reduce anemia among and improve IYCF from six mo of age |
| Loechl et al, 2009 | Haiti | Interviews and FGDs | Caregivers (mothers) | 254 | Government Sprinkle distribution along with take-home food rations for two mo from six mo of age |
| McLean et al, 2018 | Rwanda | FGDs, interviews and surveys | Mothers, grandmothers, and fathers | 1,589 | MNPs from six mo of age |
| Pelto et al, 2018 | Ethiopia | IDIs | Mothers and one grandmother | 45 | MNPs from six mo of age |
| Phuka et al, 2011 | Malawi | Ratings and FGDs | Mothers | 67 | Three new LNS vs Nutributter from eight mo of age |
| Roschnik et al, 2019 | Malawi | FGDs and IDIs | Mothers and fathers | 159 | Community led MNP from six mo of age alongside childhood development interventions and malaria chemoprevention |
| Rothman et al, 2015 | South Africa | Ratings and FGDs | Mothers | 42 | Two novel SQ-LNS from six mo of age |
| Ruel-Bergeron et al, 2018 | Malawi | IDIs and FGDs | Mothers, fathers, and grandmothers | 49 | A social and behaviour change communications campaign promoting optimal IYCF and hygiene practices, and monthly provision of daily SQ-LNS (Nutributter) from six mo of age |
| Samuel et al, 2020 | Ethiopia | Interviews | Mothers | 11 | MNPs from six mo of age |
| Shaari et al, 2019 | Malaysia | Interviews | Caregiver | 25 | Multiple micronutrient supplements from six mo of age |
| Tumilowicz et al, 2019 | Ethiopia | Survey | Mothers | 1,916 | MNP and IYCF behaviour change communication from six months of age |
| Uti et al, 2005 | Nigeria | Interviews using a questionnaire | Mothers | 100 | Vitamin A supplementation programme from one mo of age |
| Young et al, 2018 | India | Surveys and IDIs | Mothers | 120 | Iron folic acid syrup and MNPs from six mo of age |

**Extended Datafile 3b.** Acceptability of studies included in this review

| **Reference** | **Low acceptability** | **High acceptability** | **Both cited** | **Inconclusive** |
| --- | --- | --- | --- | --- |
| Aaron et al, 2011 |  | 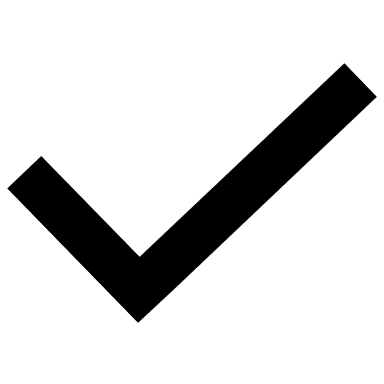 |  |  |
| Adams et al, 2018 |  |  | 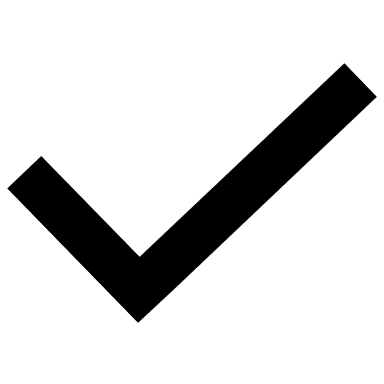 |  |
| Ahmed et al, 2014 |  |  | 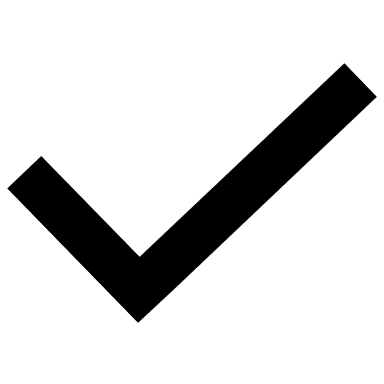 |  |
| Andersen et al, 2009 |  |  | 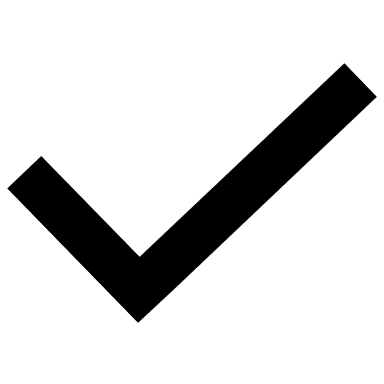 |  |
| Angdembe et al, 2015 |  | 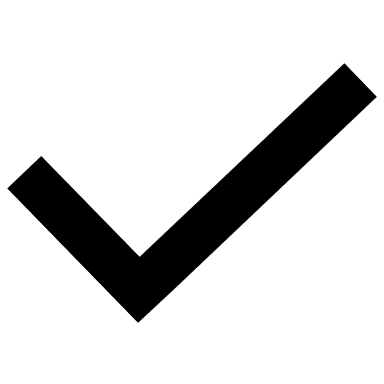 |  |  |
| Ashorn et al, 2015 |  |  | 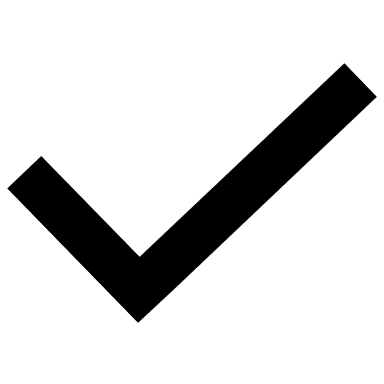 |  |
| Athavale et al, 2020 |  |  | 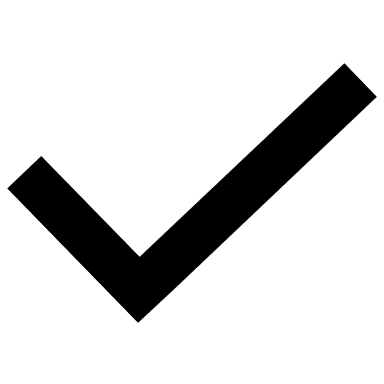 |  |
| Bashir et al, 2016 |  | 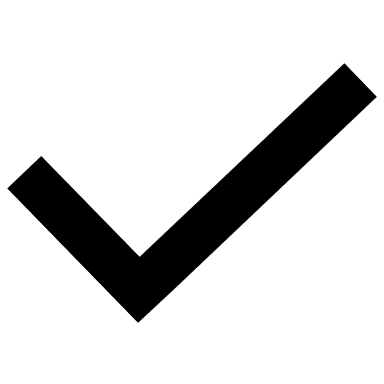 |  |  |
| Brewer et al, 2020 |  |  | 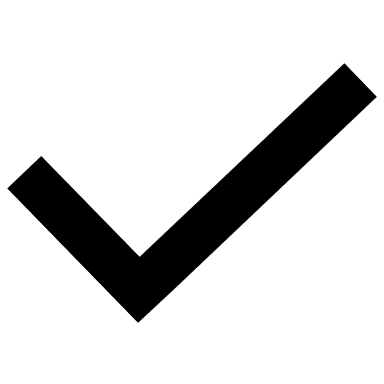 |  |
| Campos et al, 2015 |  |  |  | 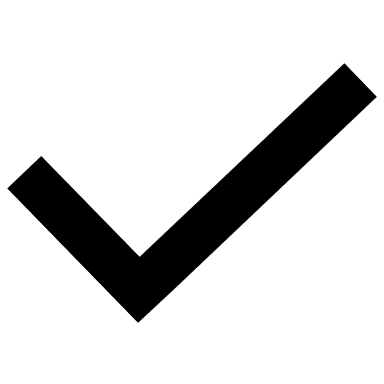 |
| Cohuet et al, 2012 |  | 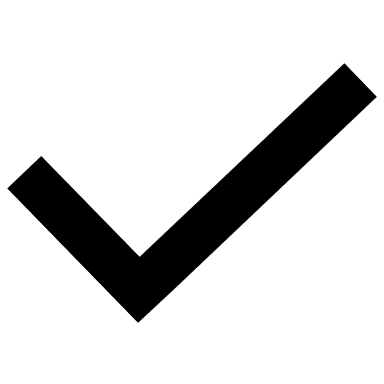 |  |  |
| Creed-Kanashiro et al, 2016 |  |  | 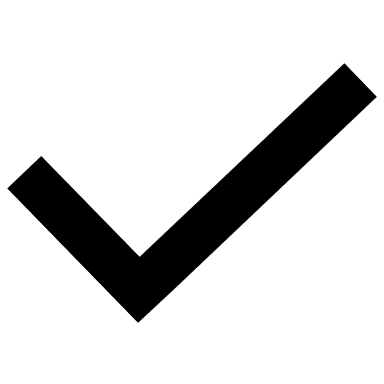 |  |
| Creed-Kanashiro et al, 2018 |  | 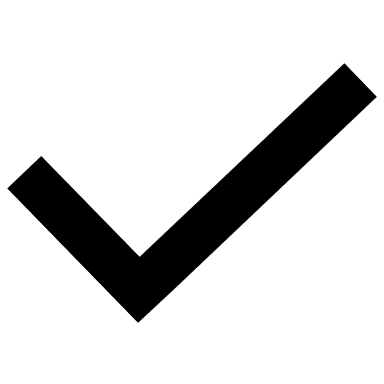 |  |  |
| Goyena et al, 2019 |  |  | 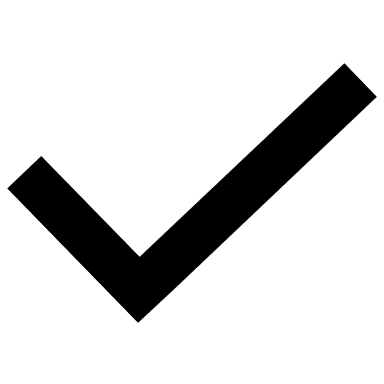 |  |
| Gunaratna et al, 2015 |  |  | 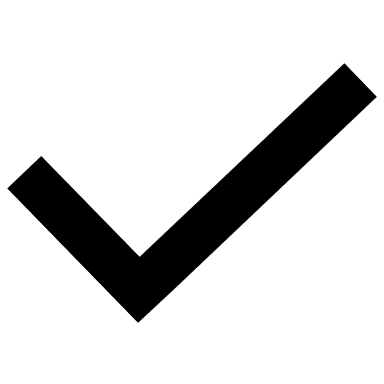 |  |
| Hess et al, 2011 |  | 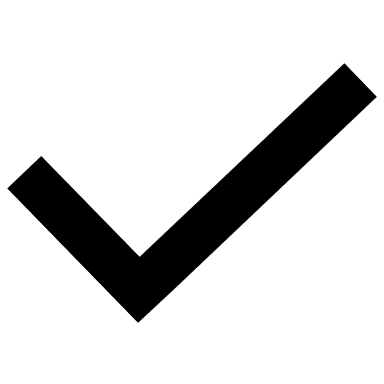 |  |  |
| Horton et al, 2018 |  |  |  | 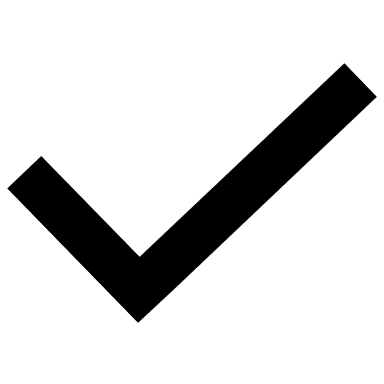 |
| Ip et al, 2009 |  | 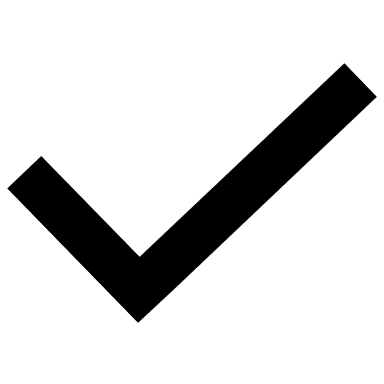 |  |  |
| Jefferds et al, 2010 |  |  | 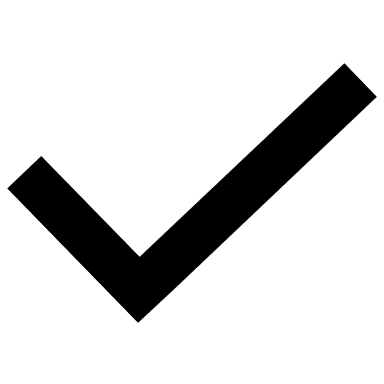 |  |
| Kajjura et al, 2020 |  |  | 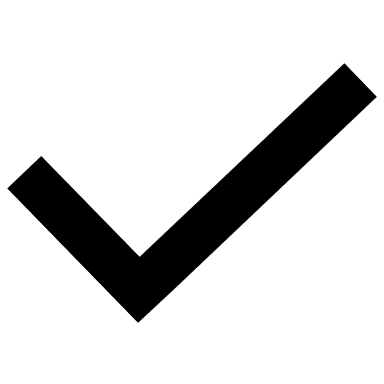 |  |
| Kodish et al, 2017 | 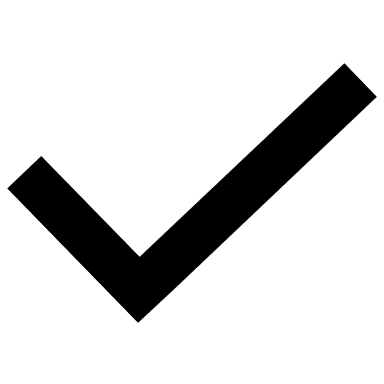 |  |  |  |
| Korenromp et al, 2015 |  |  | 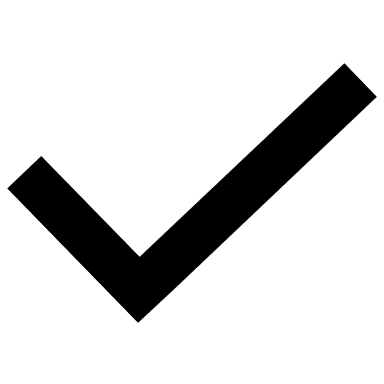 |  |
| Kung’u et al, 2015 |  | 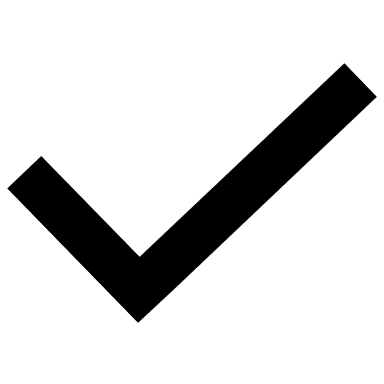 |  |  |
| Kwon et al, 2014 |  |  | 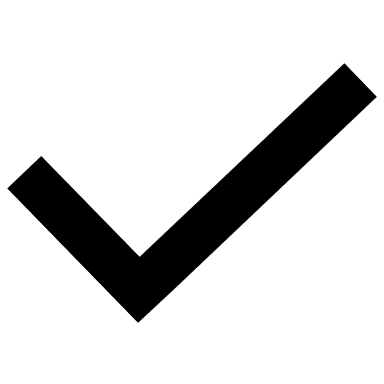 |  |
| Locks et al, 2017 |  |  | 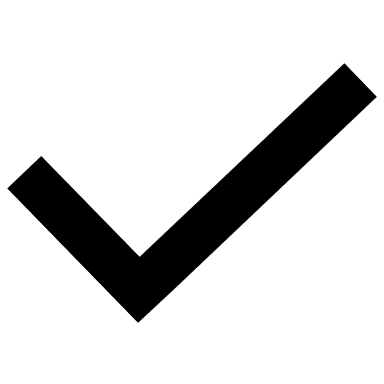 |  |
| Loechl et al, 2009 |  |  | 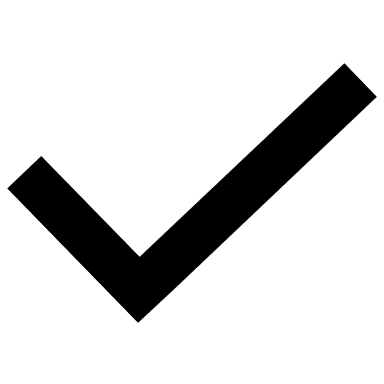 |  |
| McLean et al, 2018 |  |  | 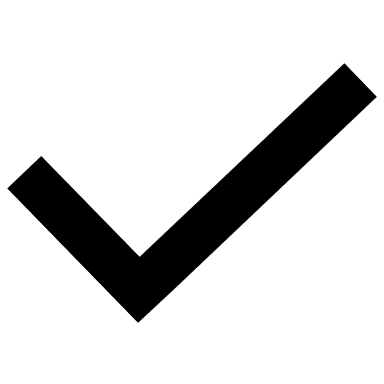 |  |
| Pelto et al, 2018 |  |  | 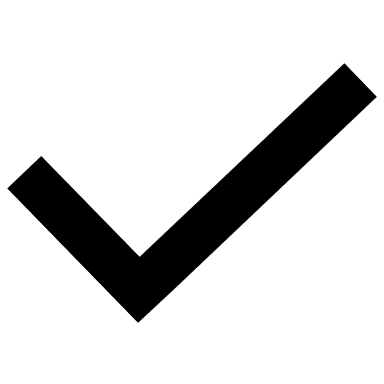 |  |
| Phuka et al, 2011 |  | 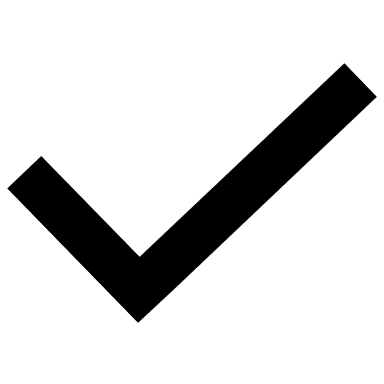 |  |  |
| Roschnik et al, 2019 |  | 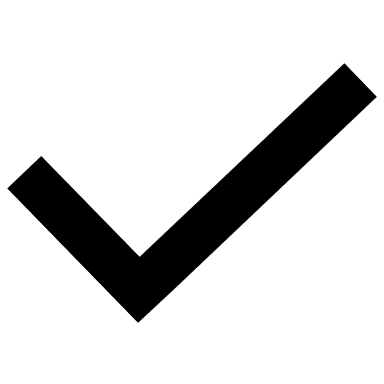 |  |  |
| Rothman et al, 2015 |  | 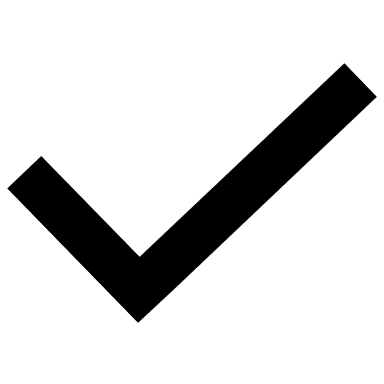 |  |  |
| Ruel-Bergeron et al, 2018 |  |  | 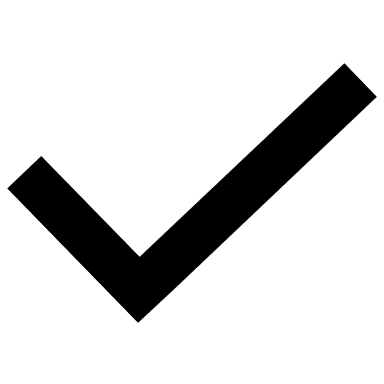 |  |
| Samuel et al, 2020 |  |  | 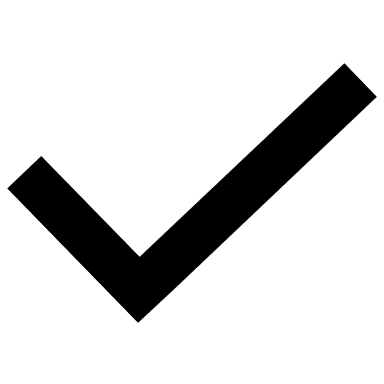 |  |
| Shaari et al, 2019 |  | 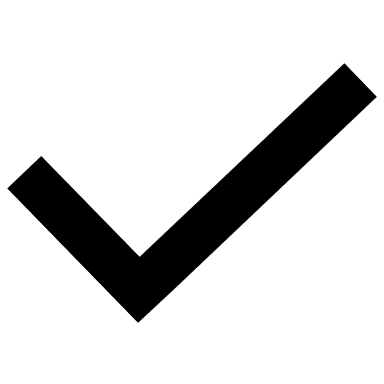 |  |  |
| Tumilowicz et al, 2019 | 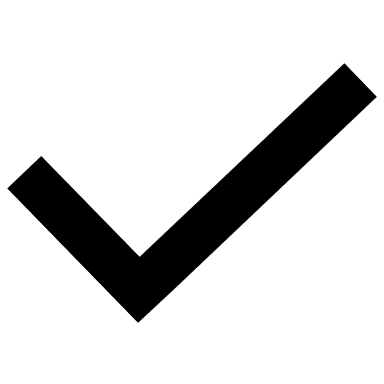 |  |  |  |
| Uti et al, 2005 |  | 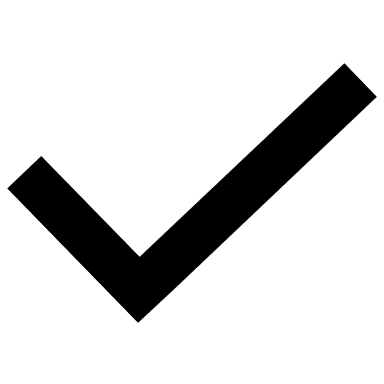 |  |  |
| Young et al, 2018 |  | 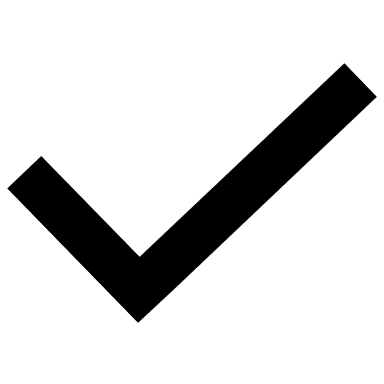 |  |  |
