# Supplementary material for: Caregiver perceptions of nutrition interventions in infants and children under 24 months of age: a systematic review
Source: Public Health Nutr. 2023 Jun 23;26(9):1907–16. doi: 10.1017/S1368980023001246 (PMC10478058; doi:10.1017/S1368980023001246)
Supplement: Supplementary file 1 [file S1368980023001246sup.zip › S1368980023001246sup002.docx]

**Extended Datafile 2: 67 Excluded Full Texts**

#22974 - Assibey-Mensah 2019

Effectiveness of Peer Counselor Support on Breastfeeding Outcomes in WIC-Enrolled Women

Assibey-Mensah, V; Suter, B; Thevenet-Morrison, K; Widanka, H; Edmunds, L; Sekhobo, J; Dozier, A

Journal of nutrition education and behavior // 2019;51(6):650-657

2019 //DOI: 10.1016/j.jneb.2019.03.005

No nutrition intervention

#23432 - Ahoya 2017

Baby friendly community initiative (BFCI): From guidelines to implementation-demonstrated results from western Kenya

Ahoya, B; Kavle, J; Gathi, C; Obade, M; Mwando, R; Akeyo, F; Straubinger, S

Annals of Nutrition and Metabolism // 2017;71(Supplement 2):527-528

B. Ahoya, Maternal Neonatal Child Health, Maternal and Child Survival Program/PATH, Kenya 2017 //DOI: 10.1159/000480486

No nutrition intervention

#21148 - Debpuur 2020

Supporting maternal and child nutrition: views from community members in rural Northern Ghana

Debpuur, C; Nonterah, E A; Chatio, S T; Adoctor, J K; Dambayi, E; Beeri, P; Nonterah, E W; Ayi-Bisah, D; Watson, D; Kehoe, S H; al., et

Public health nutrition // 2020;():1-8

2020 //DOI: 10.1017/S136898002000302X

No nutrition intervention

#21566 - deBarros 2016

Adherence to and acceptability of home fortification with vitamins and minerals in children aged 6 to 23 months: a systematic review

de Barros, S F; Cardoso, M A

BMC public health // 2016;16():299-299

2016 //DOI: 10.1186/s12889-016-2978-0

Wrong study design

#23577 - Abiona 2006

Acceptability, feasibility and affordability of infant feeding options for HIV-infected women: A qualitative study in south-west Nigeria

Abiona, T C; Onayade, A A; Ijadunola, K T; Obiajunwa, P O; Aina, O I; Thairu, L N

Maternal and Child Nutrition // 2006;2(3):135-144

T.C. Abiona, Obafemi Awolowo University, PO Box 1928, Ile-Ife 220005 Osun State, Nigeria. E-mail: titiabiona@yahoo.com 2006 //DOI: 10.1111/j.1740-8709.2006.00050.x

No nutrition intervention

#17762 - Lanou 2019

Micronutrient powder supplements combined with nutrition education marginally improve growth amongst children aged 6-23 months in rural Burkina Faso: a cluster randomized controlled trial

Lanou, H B; Osendarp, S J M; Argaw, A; De Polnay, K; Ouédraogo, C; Kouanda, S; Kolsteren, P

Maternal & child nutrition // 2019;15(4):e12820-e12820

2019 // DOI: 10.1111/mcn.12820

No caregiver perception

#22950 - Aubel 2004

Senegalese grandmothers promote improved maternal and child nutrition practices: the guardians of tradition are not averse to change

Aubel, J; Toure, I; Diagne, M

Social Science & Medicine // 2004;59(5):945-959

2004 //DOI: 10.1016/j.socscimed.2003.11.044

No nutrition intervention

#21505 - Cooper 2019

Perspectives on maternal, infant, and young child nutrition and family planning: Considerations for rollout of integrated services in Mara and Kagera, Tanzania

Cooper, C M; Kavle, J A; Nyoni, J; Drake, M; Lemwayi, R; Mabuga, L; Pfitzer, A

Maternal and Child Nutrition // 2019;15(Supplement 1):e12735-e12735

DOI: 10.1111/mcn.12735

No nutrition intervention

#13547 - Stewart 2013

Complementary feeding practices and acceptability of lipid based nutrient supplements among infants in rural western kenya

Stewart, C; Owino, V; Oiye, S; Cheyde, R; Williams, A; Dentz, H; Null, C; Dewey, K

Annals of Nutrition and Metabolism // 2013;63(SUPPL. 1):443-443

2013 //DOI: 10.1159/000354245

No full text (ie conference proceeding, abstract etc)

#11977 - Zongrone 2017

Applying a feasibility lens to an integrated nutrition program in peri-urban and urban Senegal

Zongrone, A; Le Port, A; Becquey, E; Vanderkooy, A; Huybregts, L; Diatta, A D; Sessou, E; Leroy, J; Rawat, R; Ruel, M

Annals of Nutrition and Metabolism // 2017;71(Supplement 2):789-790

2017 //DOI: 10.1159/000480486

No full text (ie conference proceeding, abstract etc)

#23425 - Aidam 2017

Building on culture: A grandmother-inclusive strategy yields significant results on maternal child health and nutrition (MCHN) outcomes in southern Sierra Leone

Aidam, B; Webb Girard, A; MacDonald, C; Wee, R; Aubel, J; Simba, J; Gruenewald, C; Bangura, A

Annals of Nutrition and Metabolism // 2017;71(Supplement 2):1244-1244

2017 //

DOI: 10.1159/000480486

No full text (ie conference proceeding, abstract etc)

#14571 - Ruel-Bergeron 2017

Growing the evidence for nutrition programming: Perceptions and implementation of a stunting prevention program rural in Malawi

Ruel-Bergeron, J; Hurley, K; Oemcke, R; Buckland, A; Kapadia-Kundu, N; Kang, Y; Shu Fune Wu, L; Mitra, M; Phuka, J; Klemm, R; West, K; Christian, P

Annals of Nutrition and Metabolism // 2017;71(Supplement 2):86-86

2017 // DOI: 10.1159/000480486

No full text (ie conference proceeding, abstract etc)

#18036 - Kim 2015

Assessing implementation fidelity of a community-based infant and young child feeding intervention in Ethiopia identifies delivery challenges that limit reach to communities: a mixed-method process evaluation study

Kim, S S; Ali, D; Kennedy, A; Tesfaye, R; Tadesse, A W; Abrha, T H; Rawat, R; Menon, P

BMC public health // 2015;15():316-316

2015 //DOI: 10.1186/s12889-015-1650-4

No nutrition intervention

#22101 - Brannon 2017

Integrating themes, evidence gaps, and research needs identified by workshop on iron screening and supplementation in iron-replete pregnant women and young children

Brannon, Patsy M; Stover, Patrick J; Taylor, Christine L

American Journal of Clinical Nutrition // 2017;106():1703S-1712S

2017 //DOI: 10.3945/ajcn.117.156083

No caregiver perception

#18490 - Kingori 2010

'Rumours' and clinical trials: a retrospective examination of a paediatric malnutrition study in Zambia, southern Africa

Kingori, P; Muchimba, M; Sikateyo, B; Amadi, B; Kelly, P; Kingori, Patricia; Muchimba, Maureen; Sikateyo, Bornwell; Amadi, Beatrice; Kelly, Paul

BMC Public Health // 2010;10(1):556-556

2010 //DOI: 10.1186/1471-2458-10-556

Wrong age group

#12961 - Ware 2018

Perceptions and experiences of caregivers of severely malnourished children receiving inpatient care in Malawi: An exploratory study

Ware, S G; Daniel, A I; Bandawe, C; Mulaheya, Y P; Nkunika, S; Nkhoma, D; Kokota, D; Stewart, R C; Voskuijl, W

Malawi medical journal : the journal of Medical Association of Malawi // 2018;30(3):167-173

2018 //DOI: 10.4314/mmj.v30i3.7

No nutrition intervention

#11845 - Sule 2009

Impact of nutritional education on nutritional status of under-five children in two rural communities of south-west Nigeria.

Sule, S S; Onayade, A A; Abiona, T C; Fatusi, A O; Ojofeitimi, E O; Esimai, O A; Ijadunola, K T

The Nigerian postgraduate medical journal // 2009;16(2):115-125

Nigeria 2009 //

No nutrition intervention

#20571 - Gonzalez 2017

Acceptability of unsweetened small quantity lipid-based nutrient supplement (SQ-LNS) in children aged 6 to 23 months: Formative research for the spoon project in Colombia, Guatemala and Mexico

Gonzalez, W; Bonvecchio, A; Duque, T; Bernal, I J; Montenegro-Bethancourt, G; Alvarado, R; Armendariz, G; Cosio, I; Vossenaar, M; Tumilowicz, A

Annals of Nutrition and Metabolism // 2017;71(Supplement 2):555-556

2017 // DOI: 10.1159/000480486

No full text (ie conference proceeding, abstract etc)

#21318 - Daly 2013

Formative research for the development of a home fortification programme for children in Zambia

Daly, Z; Suter, M; Aongola, A; McLean, J

Annals of Nutrition and Metabolism // 2013;63(SUPPL. 1):993-993

2013 //DOI: 10.1159/000354245

No full text (ie conference proceeding, abstract etc)

#23500 - Affleck 2012

Caregivers' responses to an intervention to improve young child feeding behaviors in rural Bangladesh: a mixed method study of the facilitators and barriers to change

Affleck, W; Pelto, G

Social science & medicine (1982) // 2012;75(4):651-658

2012 //DOI: 10.1016/j.socscimed.2012.03.030

No nutrition intervention

#19605 - Hellmann 2013

Withdrawal of artificial nutrition and hydration in the Neonatal Intensive Care Unit: parental perspectives

Hellmann, J; Williams, C; Ives-Baine, L; Shah, P S

Archives of Disease in Childhood -- Fetal & Neonatal Edition // 2013;98(1):F21-5

2013 //DOI: 10.1136/fetalneonatal-2012-301658

No nutrition intervention

#13389 - Suryantan 2013

Experiences from a comprehensive community nutrition program in two districts in kenya. Lessons for nationwide mnp supplementation roll out

Suryantan, J; Wambua, F; Mutua, S; Tyler, D; Bloem, M A

Annals of Nutrition and Metabolism // 2013;63(SUPPL. 1):856-856

2013 // DOI: 10.1159/000354245

No full text (ie conference proceeding, abstract etc)

#23424 - Aidam 2020

An innovative grandmother-inclusive approach for addressing suboptimal infant and young child feeding practices in Sierra Leone

Aidam, B A; MacDonald, C A; Wee, R; Simba, J; Aubel, J; Reinsma, K R; Girard, A W

Current Developments in Nutrition // 2020;4(12):nzaa174-nzaa174

2020 // DOI: 10.1093/cdn/nzaa174

No nutrition intervention

#23078 - Armendariz 2017

Knowledge, attitudes and practices of first level health workers towards breastfeeding, complementary feeding and micronutrients supplements in Mexico: Formative research for the spoon project

Armendariz, G; Alvarado, R; Bonvecchio, A; Gonzalez, W

Annals of Nutrition and Metabolism // 2017;71(Supplement 2):555-555

2017 //DOI: 10.1159/000480486

No full text (ie conference proceeding, abstract etc)

#23079 - Armar-Klemesu 2018

Using Ethnography to Identify Barriers and Facilitators to Optimal Infant and Young Child Feeding in Rural Ghana: Implications for Programs

Armar-Klemesu, Margaret; Osei-Menya, Sarah; Zakariah-Akoto, Sawudatu; Tumilowicz, Alison; Lee, James; Hotz, Christine

Food & Nutrition Bulletin // 2018;39(2):231-245

2018 // DOI: 10.1177/0379572117742298

No nutrition intervention

#23101

Breastfeeding in Samoa: A Study to Explore Women's Knowledge and the Factors which Influence Infant Feeding Practices

Archer, Lucy E; Dunne, Thomas F; Lock, Lauren J; Price, Lucy A; Ahmed, Zubair

Hawai'i journal of medicine & public health: a journal of Asia Pacific Medicine & Public Health ;76(1):15-22

No nutrition intervention

#23386 - Alam 2020

"In the past, the seeds i planted often didn't grow." A mixed-methods feasibility assessment of integrating agriculture and nutrition behaviour change interventions with cash transfers in rural Bangladesh

Alam, A; Khatun, W; Khanam, M; Ara, G; Bokshi, A; Li, M; Dibley, M J

International Journal of Environmental Research and Public Health // 2020;17(11):1-15

2020 //DOI: 10.3390/ijerph17114153

Wrong age group

#22626

Mothers' Understanding of Infant Feeding Guidelines and Their Associated Practices: A Qualitative Analysis

Begley, Andrea; Ringrose, Kyla; Giglia, Roslyn; Scott, Jane

International journal of environmental research and public health ;16(7):

DOI: 10.3390/ijerph16071141

No nutrition intervention

#23503 - Adugna 2014

Women's perception and risk factors for delayed initiation of breastfeeding in Arba Minch Zuria, Southern Ethiopia

Adugna, Dessalegn Tamiru

International Breastfeeding Journal // 2014;9(1):1-14

Nutrition Unit, Public Health Department, Arba Minch University, Arba Minch,Ethiopia 2014 //

DOI: 10.1186/1746-4358-9-8

No nutrition intervention

#23144 - Andrews 2015

'I didn't know why you had to wait': an evaluation of NHS infant-feeding workshops amongst women living in areas of high deprivation

Andrews, E J; Symon, A; Anderson, A S

Journal of Human Nutrition & Dietetics // 2015;28(6):558-567

Centre for Public Health Nutrition Research, Ninewells Hospital & Medical School, University of Dundee, Dundee UK School of Nursing & Midwifery, University of Dundee, Dundee UK 2015 // DOI: 10.1111/jhn.12269

No nutrition intervention

#12284 - Balogun 2015

Factors influencing breastfeeding exclusivity during the first 6 months of life in developing countries: A quantitative and qualitative systematic review

Balogun, Olukunmi Omobolanle; Dagvadorj, Amarjagal; Anigo, Kola Mathew; Ota, Erika; Sasaki, Satoshi

Maternal and Child Nutrition // 2015;11(4):433-451

2015 //DOI: 10.1111/mcn.12180

Wrong study design

#23464 - Ahishakiye 2017

Stressors and coping strategies of infant and young child feeding practices in Rwanda: Perceptions of mothers, fathers, grandmothers and community health workers

Ahishakiye, J; Bouwman, L; Brouwer, I; Matsiko, E; Koelen, M

Annals of Nutrition and Metabolism // 2017;71(Supplement 2):400-401

J. Ahishakiye, Dietetics Department, University of Rwanda, Rwanda 2017 //DOI: 10.1159/000480486

No full text (ie conference proceeding, abstract etc)

#18426 - Kodish 2017

Formative research to understand household utilization of a lipid-based nutrient supplement in rural Malawi and Mozambique

Kodish, Stephen

Dissertation Abstracts International: Section B: The Sciences and Engineering // 2017;78(6-B(E)):No-Specified

2017 //

No full text (ie conference proceeding, abstract etc)

#19369 - Harding 2014

Caregiver perceptions, practices, and preferences relating to multiple micronutrient powders for children 6-23 mo of age in the northern highlands of Peru

Harding, K; Zavaleta, N; Roche, M; Neufeld, L

FASEB journal // 2014;28(1 SUPPL. 1):

2014 //

No full text (ie conference proceeding, abstract etc)

#23545 - Ackatia-Armah 2016

Fostering reflective trust between mothers and community health nurses to improve the effectiveness of health and nutrition efforts: An ethnographic study in Ghana, West Africa

Ackatia-Armah, Nana M; Addy, Nii Antiaye; Ghosh, Shibani; Dubé, Laurette

Social Science & Medicine // 2016;158():96-104

McGill Center for the Convergence in Health and Economics (MCCHE), Desautels Faculty of Management, McGill University, Montreal, QC, Canada Nevin Scrimshaw International Nutrition Foundation, Friedman School of Nutrition Science and Policy, Tufts Universi 2016 // DOI: 10.1016/j.socscimed.2016.03.038

No nutrition intervention

#23484 - Agbozo 2018

Understanding why child welfare clinic attendance and growth of children in the nutrition surveillance programme is below target: lessons learnt from a mixed methods study in Ghana

Agbozo, Faith; Colecraft, Esi; Jahn, Albrecht; Guetterman, Timothy

BMC Nursing // 2018;17(1):N.PAG-N.PAG

Department of Family and Community Health, School of Public Health University of Health and Allied Sciences PMG 31 Ho Ghana Institute of Public Health University of Heidelberg Medical Faculty Heidelberg Germany Department of Nutrition and Food Science Uni 2018 //DOI: 10.1186/s12912-018-0294-y

No nutrition intervention

#23460 - Ahluwalia 2000

Georgia's breastfeeding promotion program for low-income women

Ahluwalia, I B; Tessaro, I; Grummer-Strawn, L M; MacGowan, C; Benton-Davis, S

Pediatrics // 2000;105(6):E85-E85

I.B. Ahluwalia, Division of Reproductive Health, National Center for Chronic Disease Prevention and Health Promotion, Centers for Disease Control and Prevention, Atlanta, GA 30341-3724, USA. 2000

No nutrition intervention

#21451 - Creed-Kanashiro 2017

Acceptability of multi-micronutrient powders (MNP) in young children: Factors affecting caregivers' decisions - By family, health personnel and community actors in three regions of Peru

Creed-Kanashiro, H; Bartolini, R; Goya, C; Jimenez, M

Annals of Nutrition and Metabolism // 2017;71(Supplement 2):842-842

H. Creed-Kanashiro, Instituto de Investigacion Nutricional, Lima, Peru 2017 //DOI: 10.1159/000480486

No full text (ie conference proceeding, abstract etc)

#23328 - Almeida 2020

A Qualitative Study of Breastfeeding and Formula-Feeding Mothers' Perceptions of and Experiences in WIC

Almeida, Rebeca; Alvarez Gutierrez, Shawnee; Whaley, Shannon E; Ventura, Alison K

Journal of Nutrition Education & Behavior // 2020;52(6):615-625

Department of Kinesiology and Public Health, California Polytechnic State University, San Luis Obispo, CA Division of Research and Evaluation, Public Health Foundation Enterprises WIC, Irwindale, CA 2020 //DOI: 10.1016/j.jneb.2019.12.006

No nutrition intervention

#23463 - Ahishakiye 2019

Challenges and responses to infant and young child feeding in rural Rwanda: a qualitative study

Ahishakiye, Jeanine; Bouwman, Laura; Brouwer, Inge D; Matsiko, Eric; Armar-Klemesu, Margaret; Koelen, Maria

Journal of Health, Population & Nutrition // 2019;38(1):1-10

Health and Society Chair Group, Wageningen University, Wageningen, The Netherlands Human Nutrition and Dietetics Department, College of Medicine and Health Sciences, University of Rwanda, Kigali, Rwanda Nutrition and health over the life course chair grou 2019 // DOI: 10.1186/s41043-019-0207-z

No nutrition intervention

#19155 - Ho 2013

Formative work on complementary feeding practices and anemia knowledge to guide the development of a home-fortification protocol in Rwanda

Ho, K; McLean, J; Omwega, A; Collison, D; Nyirahabineza, A; Ngabo, F

Annals of Nutrition and Metabolism // 2013;63(SUPPL. 1):654-654

K. Ho, Faculty of Land and Food Systems, University of British Columbia, Vancouver, BC, Canada 2013 //DOI: 10.1159/000354245

No full text (ie conference proceeding, abstract etc)

#23547 - Acheampong 2020

Breastfeeding and caring for children: A qualitative exploration of the experiences of mothers with physical impairments in Ghana

Acheampong, A K; Aziato, L; Marfo, M; Amevor, P

BMC Pregnancy and Childbirth // 2020;20(1):331-331

L. Aziato, Department of Adult Health, School of Nursing and Midwifery, University of Ghana, Legon, Accra, Ghana. E-mail: aziatol@yahoo.com 2020 //DOI: 10.1186/s12884-020-03028-1

No nutrition intervention

#23602 - Abbeddou 2015

Comparison of methods to assess adherence to small-quantity lipid-based nutrient supplements ( SQ- LNS) and dispersible tablets among young Burkinabé children participating in a community-based intervention trial

Abbeddou, Souheila; Hess, Sonja Y; Yakes Jimenez, Elizabeth; Somé, Jérôme W; Vosti, Stephen A; Guissou, Rosemonde M; Ouédraogo, Jean‐Bosco; Brown, Kenneth H

Maternal & Child Nutrition // 2015;11():90-104

Program in International and Community Nutrition, Department of Nutrition, University of California, Davis California, USA Nutrition/Dietetics Program, Departments of Individual, Family and Community Education and Family and Community Medicine, University 2015 //DOI: 10.1111/mcn.12162

No caregiver perception

#23007 - Bentley 2013

The breastfeeding, antiretrovirals, and nutrition (BAN) study in Malawi: use of qualitative methods to guide study design and evaluation of a randomized controlled trial

Bentley, M; Corneli, A; Parker, M; Chasela, C; Kayira, D; Moses, A; Tembo, M; Van Der Horst, C; Jamieson, D; Adair, L

Annals of nutrition & metabolism // 2013;63():25-26

2013 //DOI: 10.1159/000354245

No full text (ie conference proceeding, abstract etc)

#16322 - Muraya 2017

"If it's issues to do with nutrition…I can decide…": gendered decision-making in joining community-based child nutrition interventions within rural coastal Kenya

Muraya, Kelly W; Jones, Caroline; Berkley, James A; Molyneux, Sassy

Health Policy & Planning // 2017;32():v31-v39

Health Systems & Research Ethics Department, P.O Box 230-80108, Kilifi, Kenya Centre for Tropical Medicine & Global Health, Nuffield Department of Medicine, University of Oxford, Old Road Campus, Headington, Oxford OX3 7BN, UK 2017 // DOI: 10.1093/heapol/czx032

No caregiver perception

#15783 - Pelto 2017

The mother-child dyad is a central factor in point-of-use fortification of complementary foods with micronutrient powders in Ethiopia and Mozambique

Pelto, G; Tumilowicz, A; Kjaer Pedersen, K; Schnefke, C H; Vettersand, J; Possolo, E; Hagos, S

Annals of Nutrition and Metabolism // 2017;71(Supplement 2):533-533

G. Pelto, Division of Nutritional Sciences, Cornell University, Ithaca, NY, United States 2017 //DOI: 10.1159/000480486

No full text (ie conference proceeding, abstract etc)

#23592 - Abdollahi 2014

Oral zinc supplementation positively affects linear growth, but not weight, in children 6-24 months of age

Abdollahi, M; Abdollahi, Z; Fozouni, F; Bondarianzadeh, D

International Journal of Preventive Medicine // 2014;5(3):280-286

D. Bondarianzadeh, 7 Hafezi Street, Farahzadi Blvd, Shahrak-e-Qods, Tehran, Iran, Islamic Republic of. E-mail: d.bondarianzadeh@nnftri.ac.ir 2014 //

No caregiver perception

#23421 - Aiga 2017

Knowledge, attitude and practices: assessing maternal and child health care handbook intervention in Vietnam

Aiga, H; Nguyen, V D; Nguyen, C D; Nguyen, T T; Nguyen, L T

Tropical medicine & international health // 2017;22():329--329-

2017 // DOI: 10.1111/(ISSN)1365-3156

No nutrition intervention

#12121 - Young 2013

Community acceptability and utilization of micronutrient powders in Bihar, India

Young, M F; Kekre, P; Verma, P; Srikantiah, S; Majumdar, A; Trehan, S; Das, A K; Chaudhuri, I; Sheth, M; Webb-Girard, A; Ramakrishnan, U; Martorell, R

FASEB Journal // 2013;27(Meeting Abstracts):

M.F. Young, Global Health, Emory University, Atlanta, GA, United States 2013 //

No full text (ie conference proceeding, abstract etc)

#19160 - Hlaing 2016

Local food-based complementary feeding recommendations developed by the linear programming approach to improve the intake of problem nutrients among 12-23-month-old Myanmar children

Hlaing, L M; Fahmida, U; Htet, M K; Utomo, B; Firmansyah, A; Ferguson, E L

British Journal of Nutrition // 2016;116(S1):S16-S26

L.M. Hlaing, Department of Public Health, National Nutrition Center, Ministry of Health, Nay Pyi Taw 100604, Myanmar. E-mail: lmhlaing78@gmail.com 2016 //DOI: 10.1017/S000711451500481X

No nutrition intervention

#16326

Effect of soy flour addition and heat-processing method on nutritional quality and consumer acceptability of cassava complementary porridges

Muoki, Penina N; de Kock, Henriette L; Emmambux, Mohammad Naushad

Journal of the science of food and agriculture ;92(8):1771-1779

DOI: 10.1002/jsfa.5545

No caregiver perception

#14461 - Salasibew 2019

The fidelity and dose of message delivery on infant and young child feeding practice and nutrition sensitive agriculture in Ethiopia: a qualitative study from the Sustainable Undernutrition Reduction in Ethiopia (SURE) programme

Salasibew, M M; Moss, C; Ayana, G; Kuche, D; Eshetu, S; Dangour, A D

Journal of health, population, and nutrition // 2019;38(1):29-29

2019 // DOI: 10.1186/s41043-019-0187-z

No nutrition intervention

#22821 - Barros 2017

Designing culturally appropriate messages and approaches for maternal, infant, and young child nutrition in mozambique: Use and adaptation of trials of improved practices

Barros, I; Picolo, M; Victor, L; Kavle, J

Annals of Nutrition and Metabolism // 2017;71(Supplement 2):1267-1267

I. Barros, Maternal, Newborn, and Child Health and Nutrition Division, Maternal and Child Survival Program/ PATH, Mozambique 2017 //DOI: 10.1159/000480486

No full text (ie conference proceeding, abstract etc)

#23387 - Alam 2017

Designing an integrated agriculture and nutrition intervention to improve maternal and child nutrition in rural Bangladesh: A formative research

Alam, A; Bokshi, A; Ara, G; Li, M; McConchie, R; Khanam, M; Dibley, M

Annals of Nutrition and Metabolism // 2017;71(Supplement 2):404-404

A. Alam, Sydney School of Public Health, University of Sydney, Sydney, Australia 2017 //DOI: 10.1159/000480486

No full text (ie conference proceeding, abstract etc)

#22986 - Ashorn 2013

Long-term acceptability of LNS (lipid-based nutrient supplements) for infants in Malawi

Ashorn, U; Phiri, N; Maleta, K; Dewey, K; Arimond, M; Ashorn, P

Annals of Nutrition and Metabolism // 2013;63(SUPPL. 1):1011-1012

U. Ashorn, Department of International Health, School of Medicine, University of Tampere, Tampere, Finland 2013 //

DOI: 10.1159/000354245

No full text (ie conference proceeding, abstract etc)

#12123 - Young 2017

Caregiver perspectives and factors associated with utilization of a home fortification of complementary foods program

Young, M; Mehta, R; Gosdin, L; Kekre, P; Verma, P; Larson, L; Webb Girard, A; Ramakrishnan, U; Srikantiah, S; Martorell, R

Annals of Nutrition and Metabolism // 2017;71(Supplement 2):539-540

M. Young, Hubert Department of Global Health, Emory University, United States 2017 //DOI: 10.1159/000480486

No full text (ie conference proceeding, abstract etc)

#23440 - Ahmed 2013

Development and acceptability of locally developed ready-to-use complementary-food-supplements (RUCFS) in urban slum settings of dhaka, bangladesh

Ahmed, T; Choudhury, N; Hossain, M I; Islam, M M; Schumacher, B; De Pee, S; Tangsuphoom, N; Muiruri, J; Fuli, R; Parveen, M; Sarker, S; West Jr, K P; Christian, P

Annals of Nutrition and Metabolism // 2013;63(SUPPL. 1):582-582

T. Ahmed, ICDDR,B, Dhaka, Bangladesh 2013 // DOI: 10.1159/000354245

No full text (ie conference proceeding, abstract etc)

#20276 - Geletu 2019

Provision of low-iron micronutrient powders on alternate days is associated with lower prevalence of anaemia, stunting, and improved motor milestone acquisition in the first year of life: A retrospective cohort study in rural Ethiopia

Geletu, A; Lelisa, A; Baye, K

Maternal and Child Nutrition // 2019;15(3):e12785-e12785

K. Baye, Center for Food Science and Nutrition, College of Natural and Computational Sciences, Addis Ababa University, Addis Ababa, Ethiopia. E-mail: kaleabbaye@gmail.com 2019 // DOI: 10.1111/mcn.12785

No caregiver perception

#12295 - Adam 2019

The Philani MOVIE study: a cluster-randomized controlled trial of a mobile video entertainment-education intervention to promote exclusive breastfeeding in South Africa.

Adam, Maya; Tomlinson, Mark; Le Roux, Ingrid; LeFevre, Amnesty E; McMahon, Shannon A; Johnston, Jamie; Kirton, Angela; Mbewu, Nokwanele; Strydom, Stacy-Leigh; Prober, Charles; Barnighausen, Till

BMC health services research // 2019;19(1):211-211

England 2019 //DOI: 10.1186/s12913-019-4000-x

No nutrition intervention

#23550 - Acharya 2020

Exploring the use of mobile health to improve community-based health and nutrition service utilization in the hills of Nepal: Qualitative study

Acharya, A; Cunningham, K; Manandhar, S; Shrestha, N; Chen, M; Weissman, A

Journal of Medical Internet Research // 2020;22(9):e17659-e17659

A. Weissman, Asia Pacific Regional Office, Family Health International 360, Tower 3, Sindhorn Building, 130-132 Wireless Road, Lumpini, Phatumwan Bangkok 10330, Thailand. E-mail: aweissman@fhi360.org 2020 // DOI: 10.2196/17659

No nutrition intervention

#17448 - Mapesa 2020

Effect of community-based nutrition on infant nutrition and associated health practices in Narok, Kenya

Mapesa, J; Meme, J; Muthamia, O

African Health Sciences // 2020;20(2):724-734

J. Mapesa, Kenya Methodist University Nairobi Campus, Public Health Human Nutrition and Dietetics, Kenya. E-mail: job.mapesa@kemu.ac.ke 2020 // DOI: 10.4314/ahs.v20i2.24

No nutrition intervention

#18536 - Jordan 2018

Nutrition education improves dietary intake and growth of young children in Malawi and Cambodia-A randomised controlled trial

Jordan, I; Reinbott, A; Kuchenbecker, J; Heil, E; Phiri, G C; Krawinkel, M

Maternal & child nutrition // 2018;14():

2018 // DOI: 10.1111/mcn.12587

No full text (ie conference proceeding, abstract etc)

#22968 - Athavale 2020

A qualitative assessment of barriers and facilitators to implementing recommended infant nutrition practices in Mumbai, India

Athavale, P; Hoeft, K; Dalal, R M; Bondre, A P; Mukherjee, P; Sokal-Gutierrez, K

Journal of health, population, and nutrition // 2020;39(1):7-7

2020 // DOI: 10.1186/s41043-020-00215-w

No nutrition intervention

#23599

Assessing the Impact of a Community-Based Health and Nutrition Education on the Management of Diarrhea in an Urban District, Cairo, Egypt

Abdel-Aziz, Shaimaa B; Mowafy, Maha A; Galal, Yasmine S

Global journal of health science ;8(2):46-55

DOI: 10.5539/gjhs.v8n2p46

No nutrition intervention

#19147 - Hoddinott 2018

Randomized control trials demonstrate that nutrition-sensitive social protection interventions increase the use of multiple-micronutrient powders and iron supplements in rural pre-school Bangladeshi children

Hoddinott, J; Ahmed, A; Roy, S

Public health nutrition // 2018;21(9):1753-1761

2018 //DOI: 10.1017/S1368980017004232

No caregiver perception

#19030 - Jansen 2018

Acceptability and accessibility of child nutrition interventions: Fathers' perspectives from survey and interview studies

Jansen, E; Harris, H; Daniels, L; Thorpe, K; Rossi, T

International Journal of Behavioral Nutrition and Physical Activity // 2018;15(1):67-67

E. Jansen, Centre for Children's Health Research, 62 Graham Street (Level 6), South Brisbane, QLD 4101, Australia. E-mail: Elena.jansen@aau.at 2018 //DOI: 10.1186/s12966-018-0702-4

Wrong age group

#22752 - Bonvecchio 2007

Maternal knowledge and use of a micronutrient supplement was improved with a programmatically feasible intervention in Mexico

Bonvecchio, A; Pelto, G H; Escalante, E; Monterrubio, E; Habicht, J P; Nava, F; Villanueva, M A; Safdie, M; Rivera, J A

Journal of nutrition // 2007;137(2):440-446

2007 // DOI: 10.1093/jn/137.2.440

No caregiver perception
