# Supplementary material for: Caregiver perceptions of nutrition interventions in infants and children under 24 months of age: a systematic review
Source: Public Health Nutr. 2023 Jun 23;26(9):1907–16. doi: 10.1017/S1368980023001246 (PMC10478058; doi:10.1017/S1368980023001246)
Supplement: Supplementary file 1 [file S1368980023001246sup.zip › S1368980023001246sup004.docx]

**Extended Datafile 4.** Quality Assessment: Critical Appraisal Skills Programme (CASP) tool

| **Reference** | **1** | **2** | **3** | **4** | **5** | **6** | **7** | **8** | **9** | **10** | **Rating** |
| --- | --- | --- | --- | --- | --- | --- | --- | --- | --- | --- | --- |
| Aaron et al, 2011 | Y* | Y | Y | CD** | Y | N*** | N | Y | Y | Y | Fair |
| Adams et al, 2018 | Y | Y | Y | Y | CD | N | Y | Y | CD | Y | Fair |
| Ahmed et al, 2014 | Y | Y | Y | CD | Y | N | Y | CD | Y | CD | Poor |
| Andersen et al, 2009 | Y | Y | Y | Y | Y | Y | Y | Y | Y | Y | Good |
| Angdembe et al, 2015 | Y | Y | Y | Y | Y | Y | Y | Y | Y | Y | Good |
| Ashorn et al, 2015 | Y | Y | Y | CD | Y | Y | Y | Y | Y | Y | Good |
| Athavale et al, 2020 | Y | Y | Y | Y | Y | Y | Y | Y | Y | CD | Good |
| Bashir et al, 2016 | Y | Y | Y | Y | CD | N | Y | CD | Y | CD | Fair |
| Brewer et al, 2020 | Y | Y | Y | Y | Y | Y | Y | Y | Y | Y | Good |
| Campos et al, 2015 | Y | Y | Y | CD | Y | N | Y | Y | Y | CD | Fair |
| Cohuet et al, 2012 | Y | Y | Y | CD | Y | Y | Y | Y | Y | CD | Good |
| Creed-Kanashiro et al, 2016 | Y | Y | Y | CD | Y | N | Y | Y | Y | Y | Good |
| Creed-Kanashiro et al, 2018 | Y | Y | Y | Y | Y | Y | Y | Y | Y | CD | Good |
| Goyena et al, 2019 | Y | Y | Y | CD | Y | Y | Y | Y | Y | Y | Good |
| Gunaratna et al, 2015 | Y | Y | Y | CD | Y | Y | Y | Y | Y | Y | Good |
| Hess et al, 2011 | Y | Y | Y | CD | Y | N | Y | Y | Y | CD | Fair |
| Horton et al, 2018 | Y | Y | Y | CD | Y | Y | Y | Y | Y | CD | Good |
| Ip et al, 2009 | Y | Y | Y | CD | CD | Y | Y | CD | Y | Y | Fair |
| Jefferds et al, 2010 | Y | Y | Y | Y | Y | Y | Y | Y | Y | Y | Good |
| Kajjura et al, 2020 | Y | Y | Y | Y | Y | Y | Y | Y | Y | Y | Good |
| Kodish et al, 2917 | Y | Y | Y | CD | CD | N | Y | Y | Y | Y | Fair |
| Korenromp et al, 2015 | Y | Y | Y | Y | Y | N | Y | Y | Y | Y | Good |
| Kung’u et al, 2015 | Y | Y | Y | Y | Y | N | Y | Y | Y | CD | Good |
| Kwon et al, 2014 | Y | Y | Y | Y | CD | N | Y | Y | Y | CD | Fair |
| Locks et al, 2017 | Y | Y | Y | Y | Y | N | Y | CD | Y | Y | Good |
| Loechl et al, 2009 | Y | Y | Y | CD | CD | Y | Y | Y | Y | Y | Good |
| McLean et al, 2018 | Y | Y | Y | CD | Y | Y | Y | Y | Y | Y | Good |
| Pelto et al, 2018 | Y | Y | Y | Y | CD | N | Y | Y | Y | Y | Good |
| Phuka et al, 2011 | Y | Y | Y | Y | CD | N | Y | Y | Y | Y | Good |
| Roschnik et al, 2019 | Y | Y | Y | CD | CD | N | Y | Y | Y | Y | Good |
| Rothman et al, 2015 | Y | Y | Y | CD | Y | Y | Y | Y | Y | Y | Good |
| Ruel-Bergeron et al, 2018 | Y | Y | Y | Y | Y | Y | Y | Y | Y | Y | Good |
| Samuel et al, 2020 | Y | Y | Y | Y | CD | N | Y | Y | Y | Y | Good |
| Shaari et al, 2019 | Y | Y | Y | CD | CD | N | Y | Y | Y | CD | Fair |
| Tumilowicz et al, 2019 | Y | Y | Y | CD | CD | N | Y | CD | Y | Y | Fair |
| Uti et al, 2005 | Y | Y | Y | CD | CD | CD | Y | Y | Y | Y | Fair |
| Young et al, 2017 | Y | Y | Y | CD | CD | N | Y | Y | Y | Y | Fair |

**Y – Yes, **N – No, **CD – Cannot determine/ cannot tell*

| **Reference:** Aaron et al 2011 | **Rating** | **Description** |
| --- | --- | --- |
| 1. Was there a clear statement of the aims of the research? | Yes | To assess the acceptability of zinc-fortified, cereal-based complementary foods and zinc-fortified wheat breads |
| 2. Is a qualitative methodology appropriate? | Yes | Understanding caregiver and parents’ acceptability of a nutrition intervention in young children |
| 3. Was the research design appropriate to address the aims of the research? | Yes | Acceptability, triangle, and threshold tests |
| 4. Was the recruitment strategy appropriate to the aims of the research? | Cannot tell | Does not specify how participants were recruited |
| 5. Was the data collected in a way that addressed the research issue? | Yes | Conducted at health centres |
| 6. Has the relationship between researcher and participants been adequately considered? | No | No mention |
| 7. Have ethical issues been taken into consideration? | No | No mention |
| 8. Was the data analysis sufficiently rigorous? | Yes | Probability of type I error/ α <0.05 and power ≥0.80 for all components of the study |
| 9. Is there a clear statement of findings? | Yes | Zinc fortification of cereal flours in the ranges of fortification that were tested does not adversely affect the acceptability of complementary foods and breads prepared from these flours |
| 10. How valuable is the research? | Yes | Summaries acceptability of fortified foods |
| Comments on key limitations | The study did summarise acceptability of fortified foods, however not in qualitative outcomes (IDIs/FGDs) | |

| **Reference:** Adams et al 2018 | **Rating** | **Description** |
| --- | --- | --- |
| 1. Was there a clear statement of the aims of the research? | Yes | Retrospective and current parental perceptions  of nutrient supplements generally and of small quantity lipid based nutrient supplements (SQ‐LNS) and their effects compared with perceptions IFA and MMN capsules |
| 2. Is a qualitative methodology appropriate? | Yes | Parental perceptions of a past nutrition intervention in young children |
| 3. Was the research design appropriate to address the aims of the research? | Yes | Survey‐based methods |
| 4. Was the recruitment strategy appropriate to the aims of the research? | Yes | Pregnant women on a rolling basis due for routine visits |
| 5. Was the data collected in a way that addressed the research issue? | Cannot tell | Does not specify where the follow up study took place |
| 6. Has the relationship between researcher and participants been adequately considered? | No | No mention |
| 7. Have ethical issues been taken into consideration? | Yes | Ethics statement of approval through committee |
| 8. Was the data analysis sufficiently rigorous? | Cannot tell | No specification on qualitative data analysis |
| 9. Is there a clear statement of findings? | Yes | Almost all (≥90%) of mothers and fathers perceived that the assigned supplement positively impacted the child and expected continued positive impacts on the child's health and human capital into the future |
| 10. How valuable is the research? | Yes | Investigates long term acceptability of a nutrition intervention in young infancy |
| Comments on key limitations | The study was survey based rather than through FGDs/IDIs. Likewise, while it is interesting to have longer term acceptability (4-6 years later), this may impact parents’ memory | |

| **Reference:** Ahmed et al 2014 | **Rating** | **Description** |
| --- | --- | --- |
| 1. Was there a clear statement of the aims of the research? | Yes | To develop RUSF using locally  available food ingredients and test their acceptability |
| 2. Is a qualitative methodology appropriate? | Yes | Mothers’ acceptability of a nutrition intervention in young children |
| 3. Was the research design appropriate to address the aims of the research? | Yes | Interviews with mothers as well as their grading on a hedonic scale |
| 4. Was the recruitment strategy appropriate to the aims of the research? | Cannot tell | Does not specify |
| 5. Was the data collected in a way that addressed the research issue? | Yes | The data was collected in a health centre |
| 6. Has the relationship between researcher and participants been adequately considered? | No | Does not specify |
| 7. Have ethical issues been taken into consideration? | Yes | Ethics statement of approval through committee |
| 8. Was the data analysis sufficiently rigorous? | Cannot tell | Vague explanation of analysis |
| 9. Is there a clear statement of findings? | Yes | Interviews with the caregivers/mothers revealed that 18/30 (60%) and 20/30 (66%) of children preferred the rice-lentil and chickpea-based RUSFs, respectively |
| 10. How valuable is the research? | Cannot tell | Not specific enough differences |
| Comments on key limitations | The study did not give specifics on analysis and the results are too close to tell if the study was rigorous | |

| **Reference:** Andersen et al 2009 | **Rating** | **Description** |
| --- | --- | --- |
| 1. Was there a clear statement of the aims of the research? | Yes | To study mothers’ experience with the Nutrition Supplementation Programme (NSP), and assess this in relation to South Africa’s emphasis on human rights |
| 2. Is a qualitative methodology appropriate? | Yes | Mothers experience of a government nutrition program for children |
| 3. Was the research design appropriate to address the aims of the research? | Yes | FGDs with mothers of infants under five included in the NSP |
| 4. Was the recruitment strategy appropriate to the aims of the research? | Yes | Purposeful, criterion-based sampling method was used to select the participants for the FGDs |
| 5. Was the data collected in a way that addressed the research issue? | Yes | The data was collected through primary health care clinics |
| 6. Has the relationship between researcher and participants been adequately considered? | Yes | Hired moderators in the preferred language of the participants with some experience with moderating FGDs (trained by the main researcher and practised through pilot FGDs) |
| 7. Have ethical issues been taken into consideration? | Yes | Ethics statement of approval through committee |
| 8. Was the data analysis sufficiently rigorous? | Yes | Performed according to the Systematic Text  Condensation method developed by Malterud (modified version of Giorgi’s method) suited for the development of descriptions/notions related to human experience |
| 9. Is there a clear statement of findings? | Yes | Mothers expressed satisfaction with receiving the supplements, which they perceived to be nutritious. They received little/no education and lacked knowledge/skills regarding how to help children gain weight. Many mothers experienced poor communication with staff members as well as unfavourable comments/ lack of respect |
| 10. How valuable is the research? | Yes | Gives maternal perspective of a government funded nutrition program in young infants |
| Comments on key limitations | Only interviews mothers enrolled in program so acceptability may be skewed | |

| **Reference:** Angdembe et al 2015 | **Rating** | **Description** |
| --- | --- | --- |
| 1. Was there a clear statement of the aims of the research? | Yes | To assess adherence to MMNP and associated factors among children aged 6–59 months in rural Bangladesh |
| 2. Is a qualitative methodology appropriate? | Yes | For factors associated to a nutrition intervention in young children |
| 3. Was the research design appropriate to address the aims of the research? | Yes | Semi structured questionnaires with mothers |
| 4. Was the recruitment strategy appropriate to the aims of the research? | Yes | With a 95% confidence level and 10% margin of error, sample size was 59. Taking into account a nonresponse of 10%, final sample size was calculated to be 78. A one stage cluster sampling technique was used |
| 5. Was the data collected in a way that addressed the research issue? | Yes | By trained interviewers using a standardized pretested semi-structured questionnaire, drafted in English, and translated into Bangla |
| 6. Has the relationship between researcher and participants been adequately considered? | Yes | Trained interviewers in the local language |
| 7. Have ethical issues been taken into consideration? | Yes | Ethics statement of approval through committee |
| 8. Was the data analysis sufficiently rigorous? | Yes | Variables were fitted in the model in blocks: 1. Socio-demographic 2. Morbidity related 3. Mother’s perception related 4. Program related 5. Knowledge related 6. Acceptability related 7. Female health worker grade variable |
| 9. Is there a clear statement of findings? | Yes | MMNPs had high acceptability and adherence among mothers, with daily supplementation being preferred (to alternate days) |
| 10. How valuable is the research? | Yes | Maternal acceptability of MMNP in young infants |
| Comments on key limitations | Only used a questionnaire rather than IDI/FGD so more quantitative | |

| **Reference:** Ashorn et al 2015 | **Rating** | **Description** |
| --- | --- | --- |
| 1. Was there a clear statement of the aims of the research? | Yes | To determine the sustained acceptability of LNSs among 6- to 18-mo-old children in Malawi |
| 2. Is a qualitative methodology appropriate? | Yes | Acceptability of a nutrition intervention in young children |
| 3. Was the research design appropriate to address the aims of the research? | Yes | Knowledge, attitude, and practices (KAP) interviews at month 12 and 18 of the intervention and IDIs with a subset of mothers |
| 4. Was the recruitment strategy appropriate to the aims of the research? | CD | Does not specify |
| 5. Was the data collected in a way that addressed the research issue? | Yes | At homes of participants. The guides were translated into the local languages. |
| 6. Has the relationship between researcher and participants been adequately considered? | Yes | A Malawian female researcher visited the homes of the participants. Also carried out IDIs with participants who later dropped out |
| 7. Have ethical issues been taken into consideration? | Yes | Ethics statement of approval through committee |
| 8. Was the data analysis sufficiently rigorous? | Yes | Framework analysis; thematic analysis |
| 9. Is there a clear statement of findings? | Yes | The acceptability of LNS products was good but sharing of the products with family members and deviation from other feeding recommendations were frequent so individually targeted children were likely to receive less than the intended dose of the LNS |
| 10. How valuable is the research? | Yes | Maternal acceptability of LNS, but sharing is common |
| Comments on key limitations | Recruitment strategy not specified | |

| **Reference:** Athavale et al 2020 | **Rating** | **Description** |
| --- | --- | --- |
| 1. Was there a clear statement of the aims of the research? | Yes | In-depth qualitative assessment of family barriers and facilitators to implementing recommended child nutrition practices |
| 2. Is a qualitative methodology appropriate? | Yes | Barriers and facilitators to government nutrition recommendations |
| 3. Was the research design appropriate to address the aims of the research? | Yes | Maternal and maternal grandmothers’ perceptions to government nutrition interventions in young children |
| 4. Was the recruitment strategy appropriate to the aims of the research? | Yes | Purposively sampled |
| 5. Was the data collected in a way that addressed the research issue? | Yes | In participants homes |
| 6. Has the relationship between researcher and participants been adequately considered? | Yes | The interviews were conducted in the participant’s home, in the participant’s preferred language (Hindi or Marathi) |
| 7. Have ethical issues been taken into consideration? | Yes | Ethics statement of approval through committee |
| 8. Was the data analysis sufficiently rigorous? | Yes | Thematic analysis using inductive coding along with co-authors until saturation reached |
| 9. Is there a clear statement of findings? | Yes | Key barriers included: lack of nutrition knowledge and experience, receiving conflicting messages from different sources, limited social support, and poor self-efficacy for maternal decision-making. Key facilitators Ethics statement of approval through committee included: professional nutrition guidance, personal self-efficacy and empowerment, and family support |
| 10. How valuable is the research? | Cannot tell | Focuses more on facilitators/barriers than acceptability per se |
| Comments on key limitations | Greater focus on overall facilitators/barriers than acceptability | |

| **Reference:** Bashir et al 2016 | **Rating** | **Description** |
| --- | --- | --- |
| 1. Was there a clear statement of the aims of the research? | Yes | To evaluate the effectiveness and acceptability of ready to use therapeutic food (RUTF) among malnourished children in a tertiary care hospital |
| 2. Is a qualitative methodology appropriate? | Yes | To assess maternal acceptability of a nutrition intervention in young infants |
| 3. Was the research design appropriate to address the aims of the research? | Yes | Well structured questionnaires with mothers |
| 4. Was the recruitment strategy appropriate to the aims of the research? | Yes | Conducted at a tertiary care hospital |
| 5. Was the data collected in a way that addressed the research issue? | Cannot tell | Does not specify |
| 6. Has the relationship between researcher and participants been adequately considered? | No | No mention |
| 7. Have ethical issues been taken into consideration? | Yes | Ethics statement of approval through committee |
| 8. Was the data analysis sufficiently rigorous? | Cannot tell | No detail given |
| 9. Is there a clear statement of findings? | Yes | All mothers were satisfied from ready to use therapeutic foods (100%) |
| 10. How valuable is the research? | Cannot tell | Focuses more on quantitative acceptability outcomes (amount consumed) |
| Comments on key limitations | More quantitative, lacking FGDs/IDIs | |

| **Reference:** Brewer et al 2020 | **Rating** | **Description** |
| --- | --- | --- |
| 1. Was there a clear statement of the aims of the research? | Yes | To explore factors at all levels of the Social‐Ecological Model that affect MNP use and adherence in a Peruvian city with childhood anaemia rates higher than the national average |
| 2. Is a qualitative methodology appropriate? | Yes | To explore caregivers KAP towards MNPs and anaemia |
| 3. Was the research design appropriate to address the aims of the research? | Yes | IDIs and FGDs with caregivers |
| 4. Was the recruitment strategy appropriate to the aims of the research? |  | Convenience sampling from within/around health establishments selected by choosing one health centre and one health post per district, favouring those establishments with the highest percentage of children with anaemia |
| 5. Was the data collected in a way that addressed the research issue? | Yes | Health establishments |
| 6. Has the relationship between researcher and participants been adequately considered? | Yes | Local health personnel |
| 7. Have ethical issues been taken into consideration? | Yes | Ethics statement of approval through committee |
| 8. Was the data analysis sufficiently rigorous? | Yes | Thematic analysis with structural coding, cross coding, and blind double coding |
| 9. Is there a clear statement of findings? | Yes | Barriers: negative side effects (constipation,  vomiting, and diarrhoea), poor taste of MNP, lack of familial and peer support for its use, insufficient informational resources provided by the health system, and limited human resources. Facilitators: concern about the long-term effects of anaemia, support from organizations external to the health system, well‐coordinated care within the health system, and provision of resources by the MoH |
| 10. How valuable is the research? | Yes | Barriers ad facilitators for anemia prevention and control |
| Comments on key limitations | More focus on barriers and facilitators than specific care giver acceptability of intervention | |

| **Reference:** Campos et al 2015 | **Rating** | **Description** |
| --- | --- | --- |
| 1. Was there a clear statement of the aims of the research? | Yes | to develop a homemade chicken liver baby food (CLBF) that meets infant’s nutritional requirements for energy, macronutrients, sodium, and iron and to test its acceptance by infants and their mothers in a blind sensory test (ST) |
| 2. Is a qualitative methodology appropriate? | Yes | Maternal preference for a complementary food |
| 3. Was the research design appropriate to address the aims of the research? | Yes | Blind sensory test and hedonic scale |
| 4. Was the recruitment strategy appropriate to the aims of the research? | Cannot tell | Does not specify |
| 5. Was the data collected in a way that addressed the research issue? | Yes | In a basic health unit |
| 6. Has the relationship between researcher and participants been adequately considered? | No | Does not specify |
| 7. Have ethical issues been taken into consideration? | Yes | Ethics statement of approval through committee |
| 8. Was the data analysis sufficiently rigorous? | Yes | The Kappa index was used to measure the agreement between mothers’ like or dislike of liver and beef, answered in the questionnaire, and their score of the CLBF and ground beef baby food (GBBF) in the blind ST |
| 9. Is there a clear statement of findings? | Yes | Infants tried and liked the CLBF that match dietary recommendations and could help prevent iron deficiency. Mothers, on the other hand, demonstrated an ‘I don’t like it; I never tried it’ attitude |
| 10. How valuable is the research? | Cannot tell | Does not explore qualitative acceptability through IDIs/FGDs |
| Comments on key limitations | More quantitative outcomes | |

| **Reference:** Cohuet et al 2012 | **Rating** | **Description** |
| --- | --- | --- |
| 1. Was there a clear statement of the aims of the research? | Yes | Acceptability of RUSF within households in four districts of Niger during large scale preventive distributions with RUSF targeted at children 6–35 months of age |
| 2. Is a qualitative methodology appropriate? | Yes | To explore caregiver acceptability of a nutrition intervention targeted at young children |
| 3. Was the research design appropriate to address the aims of the research? | Yes | Cross sectional survey, IDIs and FGDs |
| 4. Was the recruitment strategy appropriate to the aims of the research? | Yes | Stratified villages into two strata based on accessibility defined as presence or absence of a health centre, market, water point, or main road within a 10 km radius. Villages within each stratum were selected using cluster-based sampling, proportional to population size, within the four districts with RUSF distributions. All households in the selected villages were eligible for inclusion in the study |
| 5. Was the data collected in a way that addressed the research issue? | Cannot tell | Does not specify |
| 6. Has the relationship between researcher and participants been adequately considered? | Yes | Community health workers within the same range as the caregivers |
| 7. Have ethical issues been taken into consideration? | Yes | Ethics statement of approval through committee |
| 8. Was the data analysis sufficiently rigorous? | Yes | The content of the FGDs and IDIs were analyzed using a list of codes defined before the start of data collection to include all themes related to the study objectives. To provide a richer analysis, we added additional codes for emerging themes that were not in the initial list. Both inductive and deductive codes were used |
| 9. Is there a clear statement of findings? | Yes | IDIs and FGDs confirmed the overall positive perception of RUSF among caregivers. Mothers also described improvements of children’s health and vitality after RUSF consumption |
| 10. How valuable is the research? | Cannot tell | Clear findings but more on practices rather than acceptability |
| Comments on key limitations | Does not specify where data was collected and focuses more on practices than acceptability per se | |

| **Reference:** Creed-Kanashiro et al 2016 | **Rating** | **Description** |
| --- | --- | --- |
| 1. Was there a clear statement of the aims of the research? | Yes | To explore caregiver acceptability of MNP and the role of health personnel (HP) in three regions of Peru piloting a MNP programme |
| 2. Is a qualitative methodology appropriate? | Yes | Yes, caregiver acceptability of a nutrition intervention in young children |
| 3. Was the research design appropriate to address the aims of the research? | Yes | Interviews and observations of caregivers |
| 4. Was the recruitment strategy appropriate to the aims of the research? | Cannot tell | Does not specify |
| 5. Was the data collected in a way that addressed the research issue? | Yes | At caregiver homes |
| 6. Has the relationship between researcher and participants been adequately considered? | No | Does not specify |
| 7. Have ethical issues been taken into consideration? | Yes | Ethics statement of approval through committee |
| 8. Was the data analysis sufficiently rigorous? | Yes | IDIs were transcribed and coded by themes, organized into thematic matrixes by region, type of participant and by peri-urban and rural areas. The researchers read all the matrixes, noting coincidences and differences within each region and between regions for each theme. The findings and comprehension of the data were discussed and consolidated |
| 9. Is there a clear statement of findings? | Yes | Caregivers expressed preference for MNP to ferrous sulfate, not only for the taste and ease to give to the child but also to keep/transport |
| 10. How valuable is the research? | Yes | Caregiver perfection of MNP vs standard intervention |
| Comments on key limitations | Does not specify recruitment strategy or relationship between researchers and participants. | |

| **Reference:** Creed-Kanashiro et al 2018 | **Rating** | **Description** |
| --- | --- | --- |
| 1. Was there a clear statement of the aims of the research? | Yes | To explore, through systematic formative research, the acceptability, use, and feasibility of a simple technology, commercial infant food grinders, in two rural Peruvian settings where there is delayed and low consumption of complementary foods of a thick consistency, including animal source foods (ASF) |
| 2. Is a qualitative methodology appropriate? | Yes | Maternal acceptability of a new nutrition technology in young children |
| 3. Was the research design appropriate to address the aims of the research? | Yes | FGDs, IDIs and home observations with mothers |
| 4. Was the recruitment strategy appropriate to the aims of the research? | Yes | Purposive sampling |
| 5. Was the data collected in a way that addressed the research issue? | Yes | In participant homes |
| 6. Has the relationship between researcher and participants been adequately considered? | Yes | Data were collected by trained IIN anthropologists and nutritionists. A local Quechua speaking interviewer conducted the data collection with Quechua speaking women in Incahuasi |
| 7. Have ethical issues been taken into consideration? | Yes | Ethics statement of approval through committee |
| 8. Was the data analysis sufficiently rigorous? | Yes | Team members including the senior investigators separately reviewed the transcripts and the detailed observational field notes. Themes and sub‐themes were coded, and thematic matrixes were constructed for everyone interviewed. Team members discussed emergent themes |
| 9. Is there a clear statement of findings? | Yes | The technology was highly acceptable, used by most mothers (87.8%), and led to changes in cultural perceptions, facilitating increased feeding of appropriate textures (thick purees), ASF, and MNPs |
| 10. How valuable is the research? | Cannot tell | Unclear if considered a nutrition intervention |
| Comments on key limitations | This is a technology rather than intervention | |

| **Reference:** Goyena et al 2019 | **Rating** | **Description** |
| --- | --- | --- |
| 1. Was there a clear statement of the aims of the research? | Yes | To assess and explore the acceptability and compliance to MNP and Bigas-Mongo (BigMo) CF blend among mothers/caregivers of children aged 6 to 23 months and the promotion of these products by community health workers (CHWs) |
| 2. Is a qualitative methodology appropriate? | Yes | Maternal/caregiver acceptability of a nutrition intervention in young children |
| 3. Was the research design appropriate to address the aims of the research? | Yes | IDIs with mothers |
| 4. Was the recruitment strategy appropriate to the aims of the research? | Cannot tell | Not enough detail given |
| 5. Was the data collected in a way that addressed the research issue? | Yes | Participants homes |
| 6. Has the relationship between researcher and participants been adequately considered? | Yes | CHWs |
| 7. Have ethical issues been taken into consideration? | Yes | Ethics statement of approval through committee |
| 8. Was the data analysis sufficiently rigorous? | Yes | The data were summarized by constructing frequency and percentage distributions. The contents were transcribed, coded by theme, consolidated, and discussed |
| 9. Is there a clear statement of findings? | Yes | Maternal adaptive strategies in preparing and feeding MNP to their children could affect MNP use, while non-receptiveness of mothers to integrating MNP into the child’s feeding routine, perceived side effects, and perceived unfavorable taste and smell were key factors considered to limit MNP use |
| 10. How valuable is the research? | Yes | Maternal acceptability of a MNP intervention in young children |
| Comments on key limitations | Does not give sufficient detail on recruitment | |

| **Reference:** Gunatatna et al 2015 | **Rating** | **Description** |
| --- | --- | --- |
| 1. Was there a clear statement of the aims of the research? | Yes | To assess women’s and children’s acceptance of a biofortified crop, quality protein maize (QPM), for CF in rural Ethiopia |
| 2. Is a qualitative methodology appropriate? | Yes | Maternal acceptability of a nutrition intervention targeted to young children |
| 3. Was the research design appropriate to address the aims of the research? | Yes | Questionnaires and hedonic scale |
| 4. Was the recruitment strategy appropriate to the aims of the research? | Cannot tell | Not enough detail given |
| 5. Was the data collected in a way that addressed the research issue? | Yes | In participants home |
| 6. Has the relationship between researcher and participants been adequately considered? | Yes | Local residents trained in all aspects of the study |
| 7. Have ethical issues been taken into consideration? | Yes | Ethics statement of approval through committee |
| 8. Was the data analysis sufficiently rigorous? | Yes | Scores for sensory characteristics and overall  acceptance, measured using an ordinal Likert scale, were analyzed using mixed effects ordinal logistic regression |
| 9. Is there a clear statement of findings? | Yes | Women were more than twice as likely to prefer QPM over conventional maize |
| 10. How valuable is the research? | Yes | Novel nutrition intervention for young children well accepted |
| Comments on key limitations | Does not give detail on recruitment/sampling | |

| **Reference:** Hess et al 2011 | **Rating** | **Description** |
| --- | --- | --- |
| 1. Was there a clear statement of the aims of the research? | Yes | Acceptability of LNS containing varying zinc per daily LNS dose among Burkinabe children 9–15 months old and their mothers |
| 2. Is a qualitative methodology appropriate? | Yes | Maternal acceptability of a nutrition intervention in young children |
| 3. Was the research design appropriate to address the aims of the research? | Yes | Maternal sensory reaction using a hedonic scale, triangle tests and maternal reports |
| 4. Was the recruitment strategy appropriate to the aims of the research? | Cannot tell | Does not specify |
| 5. Was the data collected in a way that addressed the research issue? | Yes | At the study unit and participants homes |
| 6. Has the relationship between researcher and participants been adequately considered? | No | Does not specify |
| 7. Have ethical issues been taken into consideration? | Yes | Ethics statement of approval through committee |
| 8. Was the data analysis sufficiently rigorous? | Yes | Data from the triangle tests were analysed using a binomial probability model at a significance level of P = 0.05 and 80% power. The maximum allowable false positive rate (α) was set at 0.05 and the maximum allowable false negative rate was set at 0.1. Using these criteria, at least 44 of 108 subjects (40.7%) would have to have identified the odd sample correctly to reject null hypothesis at a 5% level of significance |
| 9. Is there a clear statement of findings? | Yes | Study participation was a positive experience for mothers, and the consumption of LNS was perceived as very beneficial for their children |
| 10. How valuable is the research? | Cannot tell | More focus on perception than acceptability |
| Comments on key limitations | Does not specify recruitment strategy or relationship between participants and researchers. Does not focus on maternal qual outcomes (IDIs/FGDs) | |

| **Reference:** Horton et al 2018 | **Rating** | **Description** |
| --- | --- | --- |
| 1. Was there a clear statement of the aims of the research? | Yes | To compare cost, coverage, and user satisfaction between twice-yearly campaigns and routine delivery of vitamin A supplements in Senegal |
| 2. Is a qualitative methodology appropriate? | Yes | Caregiver perception of a nutrition intervention in young children |
| 3. Was the research design appropriate to address the aims of the research? | Yes | FGDs with caregivers |
| 4. Was the recruitment strategy appropriate to the aims of the research? | Cannot tell | Does not specify |
| 5. Was the data collected in a way that addressed the research issue? | Yes | In a private work site |
| 6. Has the relationship between researcher and participants been adequately considered? | Yes | The field staff had relevant degrees/ previous experience in undertaking qualitative studies |
| 7. Have ethical issues been taken into consideration? | Yes | Ethics statement of approval through committee |
| 8. Was the data analysis sufficiently rigorous? |  | Qualitative analysis was undertaken by the lead qualitative researcher, using well-accepted methodological principles. Transcripts were coded and reviewed to develop different coding systems for campaign and routine delivery. Content analysis was used to identify trends of concepts in and across individual codes |
| 9. Is there a clear statement of findings? | Yes | Some mothers prefer the administration of supplements at a health facility as it is perceived as more hygienic and involving professional health workers, but others, especially those living further away, prefer house-to-house delivery, which was the norm for the campaign mode, citing difficulty with travelling due to housework |
| 10. How valuable is the research? | Cannot tell | Only briefly touches on preference for administration in health facility verses home but no other maternal perceptions. |
| Comments on key limitations | Does not specify recruitment strategy | |

| **Reference:** Ip et al 2009 | **Rating** | **Description** |
| --- | --- | --- |
| 1. Was there a clear statement of the aims of the research? | Yes | To compare the effectiveness of daily and flexible administration of micronutrient Sprinkles on adherence and acceptability among young children in rural Bangladesh |
| 2. Is a qualitative methodology appropriate? | Yes | Maternal acceptability to a nutrition intervention in young infants |
| 3. Was the research design appropriate to address the aims of the research? | Yes | FGDs with mothers |
| 4. Was the recruitment strategy appropriate to the aims of the research? | Cannot tell | Does not specify |
| 5. Was the data collected in a way that addressed the research issue? | Cannot tell | Does not specify |
| 6. Has the relationship between researcher and participants been adequately considered? | Yes | Village based volunteer women health workers |
| 7. Have ethical issues been taken into consideration? | Yes | Ethics statement of approval through committee |
| 8. Was the data analysis sufficiently rigorous? | Cannot tell | Vague: All forms and questionnaires were manually checked for completeness, consistency, and range. Data were coded and processed |
| 9. Is there a clear statement of findings? | Yes | The adherence and acceptability to flexible administration over 4 months were found preferable to daily |
| 10. How valuable is the research? | Yes | Maternal acceptability of a nutrition intervention |
| Comments on key limitations | Does not specify how mothers were recruited for FGDs, where they took place or if the data analysis was rigorous | |

| **Reference:** Jefferds et al 2010 | **Rating** | **Description** |
| --- | --- | --- |
| 1. Was there a clear statement of the aims of the research? | Yes | To describe community members’ reactions  to and experiences using Sprinkles, with an emphasis on acceptability, utilization, and promotion |
| 2. Is a qualitative methodology appropriate? | Yes | Caregiver acceptability of a nutrition intervention in young children |
| 3. Was the research design appropriate to address the aims of the research? | Yes | FGDs with mothers and grandmothers |
| 4. Was the recruitment strategy appropriate to the aims of the research? | Yes | Purposeful sampling |
| 5. Was the data collected in a way that addressed the research issue? | Yes | In participant’s homes |
| 6. Has the relationship between researcher and participants been adequately considered? | Yes | Data were collected in Dholuo by Luo research  assistants |
| 7. Have ethical issues been taken into consideration? | Yes | Ethics statement of approval through committee |
| 8. Was the data analysis sufficiently rigorous? | Yes | Content analysis: coded for concepts, dominant themes, and variability |
| 9. Is there a clear statement of findings? | Yes | Sprinkles were highly acceptable to adults and most children; some children thought Sprinkles were sugar. All families reported positive effects, particularly increased appetite, and recommended Sprinkles; none experienced major problems. Potential barriers identified were lack of knowledge of and experience with Sprinkles, availability of Sprinkles, and cost |
| 10. How valuable is the research? | Yes | Caregiver acceptability of Sprinkles |
| Comments on key limitations | NA | |

| **Reference:** Kajjura et al 2020 | **Rating** | **Description** |
| --- | --- | --- |
| 1. Was there a clear statement of the aims of the research? | Yes | Maternal perceptions and barriers experienced during the management of children aged 6 to 23 months diagnosed with moderate acute malnutrition (MAM) using either a malted sorghum-based porridge (MSBP) or fortified corn soy blend (CSB+) as a supplementary porridge |
| 2. Is a qualitative methodology appropriate? | Yes | Maternal acceptability of a nutrition intervention |
| 3. Was the research design appropriate to address the aims of the research? | Yes | Maternal FGDs and IDIs |
| 4. Was the recruitment strategy appropriate to the aims of the research? | Yes | Purposively sampled |
| 5. Was the data collected in a way that addressed the research issue? | Yes | In a mutually chosen location |
| 6. Has the relationship between researcher and participants been adequately considered? | Yes | Conducted by trained research assistants in Lugbarati, the local language spoken by mothers. The research assistants were fluent in Lugbarati and resided in the Arua district, from where the mother–child pairs were recruited |
| 7. Have ethical issues been taken into consideration? | Yes | Ethics statement of approval through committee |
| 8. Was the data analysis sufficiently rigorous? | Yes | Independent manual coding transcripts twice to generate a preliminary coding framework, then classified into categories from which themes  Emerged. This was followed by content analysis |
| 9. Is there a clear statement of findings? | Yes | Mothers reported that they were satisfied with the use of MSBP or CSB+ to feed their children |
| 10. How valuable is the research? | Yes | Maternal perception of nutrition intervention in young infants |
| Comments on key limitations | NA | |

| **Reference:** Kodish et al 2017 | **Rating** | **Description** |
| --- | --- | --- |
| 1. Was there a clear statement of the aims of the research? | Yes | Patterns and determinants of SQ-LNS utilization among children 6–23months and potential sharing practices of other household members prior to intervention development |
| 2. Is a qualitative methodology appropriate? | Yes | Caregiver perception towards nutrition intervention in young infants |
| 3. Was the research design appropriate to address the aims of the research? | Yes | Caregiver IDIs |
| 4. Was the recruitment strategy appropriate to the aims of the research? | Cannot tell | Does not specify |
| 5. Was the data collected in a way that addressed the research issue? | Cannot tell | Does not specify |
| 6. Has the relationship between researcher and participants been adequately considered? | No | Does not specify |
| 7. Have ethical issues been taken into consideration? | Yes | Ethics statement of approval through committee |
| 8. Was the data analysis sufficiently rigorous? | Yes | Atlas.ti v7.0 was used for coding and analysis of the transcripts. Grounded theory: line by line coding, focused coding, analytic notes |
| 9. Is there a clear statement of findings? | Yes | IDIs revealed that caregivers did not provide the SQ-LNS in most cases during the full-day observations because they ran out of supply too early |
| 10. How valuable is the research? | Yes | Both maternal and paternal perception of a nutrition intervention in young infants |
| Comments on key limitations | Does not specify recruitment strategy, where data collection took place or relationship between interviewer and participants | |

| **Reference:** Korenromp et al 2015 | **Rating** | **Description** |
| --- | --- | --- |
| 1. Was there a clear statement of the aims of the research? | Yes | To determine the feasibility of distributing MNPs for home fortification during biannual Maternal, Neonatal and Child Health Week (MNCHW) events, as a strategy to improve young child nutrition |
| 2. Is a qualitative methodology appropriate? | Yes | Caregiver acceptability of a nutrition intervention in young children |
| 3. Was the research design appropriate to address the aims of the research? | Yes | Caregiver surveys |
| 4. Was the recruitment strategy appropriate to the aims of the research? | Yes | Stratified sample of caregivers at the facilities, longitudinal cohort |
| 5. Was the data collected in a way that addressed the research issue? | Yes | At health facilities or participants homes |
| 6. Has the relationship between researcher and participants been adequately considered? | No | Does not specify |
| 7. Have ethical issues been taken into consideration? | Yes | Ethics statement of approval through committee |
| 8. Was the data analysis sufficiently rigorous? | Yes | Survey data were analysed using SPSS. Differences in responses between the first and second home visits were analysed using the independent Student t test for continuous variables, the χ2 test for categorical variables and the normal distribution test of difference in proportions for proportional outcomes |
| 9. Is there a clear statement of findings? | Yes | Among caregivers who received MNP, acceptance and use among targeted children was high |
| 10. How valuable is the research? | Yes | Caregiver acceptability of a nutrition intervention in young children |
| Comments on key limitations | Does not specify relationship between researcher and participant | |

| **Reference:** Kung’u et al 2015 | **Rating** | **Description** |
| --- | --- | --- |
| 1. Was there a clear statement of the aims of the research? | Yes | whether the Maternal, Newborn and Child Health Weeks (MNCHW) would present an opportunity to raise awareness of and demand for the use of zinc and ORS in the treatment for diarrhoea |
| 2. Is a qualitative methodology appropriate? | Yes | Caregiver perception of a nutrition intervention in young infants |
| 3. Was the research design appropriate to address the aims of the research? | Yes | Caregiver interviews |
| 4. Was the recruitment strategy appropriate to the aims of the research? | Yes | Randomly selected by systematic sampling in which every kth caregiver was selected, where k=number of caregivers visiting the health clinics÷10 (the number of caregivers interviewed at each health clinic) |
| 5. Was the data collected in a way that addressed the research issue? | Yes | At health facilities or participant’s homes |
| 6. Has the relationship between researcher and participants been adequately considered? | No | Does not specify |
| 7. Have ethical issues been taken into consideration? | Yes | Ethics statement of approval through committee |
| 8. Was the data analysis sufficiently rigorous? | Yes | Thematically coded |
| 9. Is there a clear statement of findings? | Yes | Attitudes regarding the use of zinc in the treatment for diarrhoea and practices, measured by care-seeking behaviour, also improved between the pre-MNCHW interviews |
| 10. How valuable is the research? | Cannot tell | More focus on change in attitude between health visits |
| Comments on key limitations | This study focuses on change is attitude towards a health intervention rather than acceptability | |

| **Reference:** Kwon et al 2014 | **Rating** | **Description** |
| --- | --- | --- |
| 1. Was there a clear statement of the aims of the research? | Yes | Mothers’ KAP of iron deficiency anaemia and supplementation |
| 2. Is a qualitative methodology appropriate? | Yes | Maternal KAP of a nutritional deficiency and intervention in young infants |
| 3. Was the research design appropriate to address the aims of the research? | Yes | Maternal semi-structured interviews and FGDs |
| 4. Was the recruitment strategy appropriate to the aims of the research? | Yes | Purposive sampling framework |
| 5. Was the data collected in a way that addressed the research issue? | Cannot tell | Does not specify |
| 6. Has the relationship between researcher and participants been adequately considered? | No | Does not specify |
| 7. Have ethical issues been taken into consideration? | Yes | Ethics statement of approval through committee |
| 8. Was the data analysis sufficiently rigorous? | Yes | both thematic analysis23 and individual interview synthesis |
| 9. Is there a clear statement of findings? | Yes | Increasing community awareness of mild anemia, simplifying dosage instructions, and further strengthening the supportive environment for health workers would help in reducing the prevalence of IDA |
| 10. How valuable is the research? | Cannot tell | The research more focuses on barriers to successful interventions rather than maternal acceptability |
| Comments on key limitations | Does not specify who conducted the interviews or where | |

| **Reference:** Locks et al 2017 | **Rating** | **Description** |
| --- | --- | --- |
| 1. Was there a clear statement of the aims of the research? | Yes | The impact of an integrated infant and young child feeding (IYCF) and MNP intervention on children’s risk of anemia and IYCF practices |
| 2. Is a qualitative methodology appropriate? | Yes | Maternal acceptability of a nutrition intervention in young infants |
| 3. Was the research design appropriate to address the aims of the research? | Yes | Maternal surveys |
| 4. Was the recruitment strategy appropriate to the aims of the research? | Yes | Stratified multi-staged sampling |
| 5. Was the data collected in a way that addressed the research issue? | Yes | At participant’s homes |
| 6. Has the relationship between researcher and participants been adequately considered? | No | Does not specify |
| 7. Have ethical issues been taken into consideration? | Yes | Ethics statement of approval through committee |
| 8. Was the data analysis sufficiently rigorous? | Cannot tell | Does not specify the qual analysis |
| 9. Is there a clear statement of findings? | Yes | The addition of MNPs was well received and may improve IYCF |
| 10. How valuable is the research? | Yes | Maternal perception of a nutrition intervention in young children |
| Comments on key limitations | Does not specify the researcher and participant relationship or qual data analysis | |

| **Reference:** Loechl et al 2009 | **Rating** | **Description** |
| --- | --- | --- |
| 1. Was there a clear statement of the aims of the research? | Yes | The feasibility and acceptability of distributing micronutrient Sprinkles through a food-assisted  maternal and child health and nutrition programme |
| 2. Is a qualitative methodology appropriate? | Yes | Maternal acceptability of a nutrition intervention in young children |
| 3. Was the research design appropriate to address the aims of the research? | Yes | Maternal interviews and FGDs |
| 4. Was the recruitment strategy appropriate to the aims of the research? | Cannot tell | Does not specify |
| 5. Was the data collected in a way that addressed the research issue? | Cannot tell | Does not specify |
| 6. Has the relationship between researcher and participants been adequately considered? | Yes | Female village programme staff for FGDs and interviews were conducted by a consultant (external to the programme) who had ample prior experience with the use of qualitative research methods |
| 7. Have ethical issues been taken into consideration? | Yes | Ethics statement of approval through committee |
| 8. Was the data analysis sufficiently rigorous? | Yes | Transcripts were analysed separately. These data were supplemented with interviewer’s field notes, which provided information on the participants, the context of the interview and the interviewer’s own reflections about the interview |
| 9. Is there a clear statement of findings? | Yes | Mothers understood instructions about use of Sprinkles, acceptance was high, and no selling of the product was reported or observed |
| 10. How valuable is the research? | Yes | Maternal acceptability of a nutrition intervention in young children |
| Comments on key limitations | Does not specify how mothers were selected or where the interviews took place | |

| **Reference:** McLean et al 2018 | **Rating** | **Description** |
| --- | --- | --- |
| 1. Was there a clear statement of the aims of the research? | Yes | To develop and test an implementation strategy and tools for an effective MNP programme adapted to the local context and health system |
| 2. Is a qualitative methodology appropriate? | Yes | Caregiver acceptability of a nutrition intervention in young infants |
| 3. Was the research design appropriate to address the aims of the research? | Yes | Caregiver FDs, IDIs and an acceptability survey |
| 4. Was the recruitment strategy appropriate to the aims of the research? | Yes | Three‐stage cluster sampling with purposive and systematic sampling |
| 5. Was the data collected in a way that addressed the research issue? | Cannot tell | Does not specify |
| 6. Has the relationship between researcher and participants been adequately considered? | Yes | The cultural context, food availability, and local capacity were evaluated to guide operational components of the programme with communication messages and tools developed from the results |
| 7. Have ethical issues been taken into consideration? | Yes | Ethics statement of approval through committee |
| 8. Was the data analysis sufficiently rigorous? | Yes | Framework analysis: hand‐coding for smaller data sets. The data were examined with multiple iterative processes, and visualizations were used to describe dominant themes |
| 9. Is there a clear statement of findings? | Yes | Appropriate food vehicles for MNP in the local context were identified, as well as preferred communication methods, and trusted sources of information in the community. Implementation of the programme continued to pose challenges during scale‐up with issues pertaining to stock‐outs, delivery, and reporting among partners |
| 10. How valuable is the research? | Yes | Caregiver acceptability of a nutrition intervention in young children |
| Comments on key limitations | Did not specify where the IDIs and FGDs occurred | |

| **Reference:** Pelto et al 2018 | **Rating** | **Description** |
| --- | --- | --- |
| 1. Was there a clear statement of the aims of the research? | Yes | household responses to MNP interventions, with an emphasis on caregivers' perspectives |
| 2. Is a qualitative methodology appropriate? | Yes | Caregiver acceptability of a nutrition intervention in young children |
| 3. Was the research design appropriate to address the aims of the research? | Yes | IDIs with mothers and one grandmother |
| 4. Was the recruitment strategy appropriate to the aims of the research? | Yes | Purposive sampling |
| 5. Was the data collected in a way that addressed the research issue? | Cannot tell | Does not specify |
| 6. Has the relationship between researcher and participants been adequately considered? | No | Does not specify |
| 7. Have ethical issues been taken into consideration? | Yes | Ethics statement of approval through committee |
| 8. Was the data analysis sufficiently rigorous? | Yes | Thematic analysis with multiple levels and iterations of coding |
| 9. Is there a clear statement of findings? | Yes | Attention to caregivers' perspectives reflected in their narratives offers opportunities to improve MNP utilization in Ethiopia, with potential application in other social and cultural settings |
| 10. How valuable is the research? | Yes | Maternal acceptability of a MNP intervention in young infants |
| Comments on key limitations | Does not specify relationship between researcher and participant or where data was collected | |

| **Reference:** Phuka et al 2011 | **Rating** | **Description** |
| --- | --- | --- |
| 1. Was there a clear statement of the aims of the research? | Yes | Maternal acceptability of three new LNSs in 8–12-month-old healthy children and their caregivers |
| 2. Is a qualitative methodology appropriate? | Yes | Maternal acceptability of a nutrition intervention in young infants |
| 3. Was the research design appropriate to address the aims of the research? | Yes | Consumption and hedonic rating as well as maternal FGD |
| 4. Was the recruitment strategy appropriate to the aims of the research? | Yes | Purposively selected |
| 5. Was the data collected in a way that addressed the research issue? | Cannot tell | Does not specify |
| 6. Has the relationship between researcher and participants been adequately considered? | No | Does not specify |
| 7. Have ethical issues been taken into consideration? | Yes | Ethics statement of approval through committee |
| 8. Was the data analysis sufficiently rigorous? | Yes | Qualitative analysis using Atlas.ti for emerging themes |
| 9. Is there a clear statement of findings? | Yes | The novel LNS was largely accepted |
| 10. How valuable is the research? | Yes | Maternal acceptability of a novel LNS in young children |
| Comments on key limitations | Does not specify the relationship between researcher and participant or where FGDs took place | |

| **Reference:** Roschnik et al 2019 | **Rating** | **Description** |
| --- | --- | --- |
| 1. Was there a clear statement of the aims of the research? | Yes | Adherence and acceptability of a community‐led MNP intervention targeting children aged 6–59 months |
| 2. Is a qualitative methodology appropriate? | Yes | Caregiver acceptability of a nutrition intervention in young children |
| 3. Was the research design appropriate to address the aims of the research? | Yes | Caregiver FGDs and IDIs and direct observations |
| 4. Was the recruitment strategy appropriate to the aims of the research? | Cannot tell | Does not specify |
| 5. Was the data collected in a way that addressed the research issue? | Cannot tell | Does not specify |
| 6. Has the relationship between researcher and participants been adequately considered? | No | Does not specify |
| 7. Have ethical issues been taken into consideration? | Yes | Ethics statement of approval through committee |
| 8. Was the data analysis sufficiently rigorous? | Yes | Iterative content analysis |
| 9. Is there a clear statement of findings? | Yes | Likely contributors to uptake include perceived positive changes in the children following MNP use, the selection of a food vehicle that was already commonly given to children and the community driven, decentralized, and integrated delivery approach |
| 10. How valuable is the research? | Yes | Caregiver acceptability of a MNP intervention in young children |
| Comments on key limitations | Recruitment strategy for the qualitative data was not outlined, nor was relationship between researcher and participant or where FGDs/IDIs were conducted | |

| **Reference:** Rothman et al 2015 | **Rating** | **Description** |
| --- | --- | --- |
| 1. Was there a clear statement of the aims of the research? | Yes | The acceptability of 2 novel, SQ-LNS (A and B) for supplementing complementary foods among infants aged 6 to 12 months |
| 2. Is a qualitative methodology appropriate? | Yes | Maternal acceptability of a nutrition intervention in young infants |
| 3. Was the research design appropriate to address the aims of the research? | Yes | Maternal FGDs and exit interviews and hedonic rating |
| 4. Was the recruitment strategy appropriate to the aims of the research? | Cannot tell | Does not specify |
| 5. Was the data collected in a way that addressed the research issue? | Yes | Participant’s home and central field station (FGDs) |
| 6. Has the relationship between researcher and participants been adequately considered? | Yes | Fieldworkers and FGD facilitated by a trained facilitator all in the local languages |
| 7. Have ethical issues been taken into consideration? | Yes | Ethics statement of approval through committee |
| 8. Was the data analysis sufficiently rigorous? | Yes | A thematic framework was compiled by identifying themes (key words); tabulating was done by managing, cleaning, and simplifying the data; and interpretation was done by identifying main expressions |
| 9. Is there a clear statement of findings? | Yes | More than 70% of mothers reported a score ≥ 4 on sensory attributes for both SQ-LNSs indicating that both supplements were well perceived. FGDs confirmed a positive attitude toward the supplements in the study population |
| 10. How valuable is the research? | Yes | Maternal acceptability of novel SQ-LNS in young children |
| Comments on key limitations | Does not specify how participants were recruited for FGDs | |

| **Reference:** Ruel-Bergeron et al 2018 | **Rating** | **Description** |
| --- | --- | --- |
| 1. Was there a clear statement of the aims of the research? | Yes | To understand the facilitators and barriers to participation in a SQ-LNS program using IDIs, and direct observations with caretakers |
| 2. Is a qualitative methodology appropriate? | Yes | Caregiver perception of a nutrition intervention in young infants |
| 3. Was the research design appropriate to address the aims of the research? | Yes | Caregiver IDIs and direct observations |
| 4. Was the recruitment strategy appropriate to the aims of the research? | Yes | Purposive sampling for mothers and convenience for grandmothers/fathers |
| 5. Was the data collected in a way that addressed the research issue? | Yes | In participants homes |
| 6. Has the relationship between researcher and participants been adequately considered? | Yes | Village leaders and program volunteers. The purpose of separating community members into subgroups for FGDs was meant to elicit and categorize themes that might arise based on their common status and responsibilities in the community |
| 7. Have ethical issues been taken into consideration? | Yes | Ethics statement of approval through committee |
| 8. Was the data analysis sufficiently rigorous? | Yes | Grounded theory. Key themes from IDI and FGD were used to inductively create a formal codebook with definitions for each code. Line-by-line coding was conducted by a team of four standardized researchers whose consistency in coding was ensured using an interrater reliability test |
| 9. Is there a clear statement of findings? | Yes | Perceptions of the SQ-LNS were positive, and visible changes in child health contributed to program participation. Conflicting priorities that prevented monthly collection of SQ-LNS  and limited knowledge of child feeding messages constituted barriers to program participation |
| 10. How valuable is the research? | Yes | Caregiver (not only maternal but also paternal and grandmother) acceptability of a SQ-LNS intervention in young children |
| Comments on key limitations | NA | |

| **Reference:** Samuel et al 2020 | **Rating** | **Description** |
| --- | --- | --- |
| 1. Was there a clear statement of the aims of the research? | Yes | To assess factors associated with intake adherence and drivers for correct MNP use over time to inform scale-up of MNP interventions |
| 2. Is a qualitative methodology appropriate? | Yes | Maternal factors for use of MNP supplementation in young children |
| 3. Was the research design appropriate to address the aims of the research? | Yes | Maternal interviews |
| 4. Was the recruitment strategy appropriate to the aims of the research? | Yes | Mothers who were at the health post for the monthly follow up were selected randomly,  irrespective of adherence or non-adherence |
| 5. Was the data collected in a way that addressed the research issue? | Cannot tell | Does not specify |
| 6. Has the relationship between researcher and participants been adequately considered? | No | Does not specify |
| 7. Have ethical issues been taken into consideration? | Yes | Ethics statement of approval through committee |
| 8. Was the data analysis sufficiently rigorous? | Yes | The responses were coded and categorized according to the theoretical framework of the TPB |
| 9. Is there a clear statement of findings? | Yes | Free MNP provision, trust in the government  and field staff played a role in successful implementation. MNP is promising to be scaled up, by considering factors that positively and negatively determine adherence |
| 10. How valuable is the research? | Yes | Maternal acceptability of a MNP intervention in young children |
| Comments on key limitations | Does not specify where data was collected or relationship between interviewer and participant | |

| **Reference:** Shaari et al 2019 | **Rating** | **Description** |
| --- | --- | --- |
| 1. Was there a clear statement of the aims of the research? | Yes | To assess the feasibility of using MMS among Malaysian children |
| 2. Is a qualitative methodology appropriate? | Yes | Caregiver feasibility of a nutrition intervention in young children |
| 3. Was the research design appropriate to address the aims of the research? | Yes | Interviews with caregivers |
| 4. Was the recruitment strategy appropriate to the aims of the research? | Cannot tell | Does not specify |
| 5. Was the data collected in a way that addressed the research issue? | Cannot tell | Does not specify |
| 6. Has the relationship between researcher and participants been adequately considered? | No | Does not specify |
| 7. Have ethical issues been taken into consideration? | Yes | Ethics statement of approval through committee |
| 8. Was the data analysis sufficiently rigorous? | Yes | Data on compliance, acceptance, child preference and the adverse effect of MMS were expressed as frequencies and percentages |
| 9. Is there a clear statement of findings? | Yes | Caregivers had high acceptance of MMS with no adverse effects reported |
| 10. How valuable is the research? | Cannot tell | Very limited results on acceptability |
| Comments on key limitations | Does not specify how participants were recruited, where data was collected or relationship between interviewer and participant | |

| **Reference:** Uti et al 2005 | **Rating** | **Description** |
| --- | --- | --- |
| 1. Was there a clear statement of the aims of the research? | Yes | To assess performance of a trial to deliver  MNP in 6–23-month-old children |
| 2. Is a qualitative methodology appropriate? | Yes | Maternal acceptability of a nutrition in young children |
| 3. Was the research design appropriate to address the aims of the research? | Yes | Population based cross-sectional survey |
| 4. Was the recruitment strategy appropriate to the aims of the research? | Cannot tell | Does not specify |
| 5. Was the data collected in a way that addressed the research issue? | Cannot tell | Does not specify |
| 6. Has the relationship between researcher and participants been adequately considered? | No | Does not specify |
| 7. Have ethical issues been taken into consideration? | Yes | Ethics statement of approval through committee |
| 8. Was the data analysis sufficiently rigorous? | Cannot tell | Does not specify for qual outcomes |
| 9. Is there a clear statement of findings? | Yes | MNP was accepted as most positive but with the highest negative side effects in the rural populations. Urban populations found the feeding the of MNP most challenging |
| 10. How valuable is the research? | Yes | Maternal acceptability of a MNP intervention in young children |
| Comments on key limitations | Does not specify qual data analysis, how participants were recruited, where the data was collected or relationship between interviewer and participant | |

| **Reference:** Uti et al 2011 | **Rating** | **Description** |
| --- | --- | --- |
| 1. Was there a clear statement of the aims of the research? | Yes | To determine the KAPs of mothers attending the centre towards vitamin A-rich foods and oral vitamin A supplements |
| 2. Is a qualitative methodology appropriate? | Yes | Maternal KAP of a nutrition intervention in young children |
| 3. Was the research design appropriate to address the aims of the research? | Yes | Interviews with a questionnaire to determine maternal KAPs |
| 4. Was the recruitment strategy appropriate to the aims of the research? | Cannot tell | All mothers who attended clinic in a two-week window were included |
| 5. Was the data collected in a way that addressed the research issue? | Cannot tell | Does not specify |
| 6. Has the relationship between researcher and participants been adequately considered? | Cannot tell | Researcher and two trained assistants |
| 7. Have ethical issues been taken into consideration? | Yes | Ethics statement of approval through committee |
| 8. Was the data analysis sufficiently rigorous? | Yes | After checking and coding, data were transferred from the questionnaire to the spreadsheet, summary, frequencies; percentages of relative scores were calculated as appropriate, tables were also created. The data were analysed using descriptive statistics |
| 9. Is there a clear statement of findings? | Yes | Vitamin A supplements were well accepted, and mothers noted positive change in their children's health |
| 10. How valuable is the research? | Yes | Maternal acceptability of vitamin A supplements in young children |
| Comments on key limitations | Does not specify where data was collected and the recruitment strategy and relationship between participant and researcher both do not appear rigorous | |

| **Reference:** Young et al 2017 | **Rating** | **Description** |
| --- | --- | --- |
| 1. Was there a clear statement of the aims of the research? | Yes | Examine the acceptability of IFAS versus MNPs for children 6–23 months old |
| 2. Is a qualitative methodology appropriate? | Yes | Household/maternal acceptability of a nutrition intervention in young children |
| 3. Was the research design appropriate to address the aims of the research? | Yes | Household surveys and maternal IDIs |
| 4. Was the recruitment strategy appropriate to the aims of the research? | Cannot tell | Does not specify for the qualitative |
| 5. Was the data collected in a way that addressed the research issue? | Cannot tell | Does not specify |
| 6. Has the relationship between researcher and participants been adequately considered? | No | Does not specify |
| 7. Have ethical issues been taken into consideration? | Yes | Ethics statement of approval through committee |
| 8. Was the data analysis sufficiently rigorous? | Yes | Interviews were analysed using MaxQDA  and Excel employing a thematic analysis approach. Deductive themes defined a priori were coded and described, and inductive themes emerging from the data were also noted and coded |
| 9. Is there a clear statement of findings? | Yes | Mothers indicated that the direct administration of IFAS ensured that children consumed the full dose, and MNPs intake depended on the quantity of food consumed, especially among younger children, which emphasizes the need to integrate supplementation with the promotion of optimal IYCF practices |
| 10. How valuable is the research? | Yes | Maternal and household acceptability of a MNP intervention in young children |
| Comments on key limitations | Does not specify how qualitative data was collected, how participants were chosen for IDIs or the relationship between the researcher and participant | |
